# Application of the SLAPNAP statistical learning tool to broadly neutralizing antibody HIV prevention research

**DOI:** 10.1016/j.isci.2023.107595

**Published:** 2023-08-09

**Authors:** Brian D. Williamson, Craig A. Magaret, Shelly Karuna, Lindsay N. Carpp, Huub C. Gelderblom, Yunda Huang, David Benkeser, Peter B. Gilbert

**Affiliations:** 1 Biostatistics Division; Kaiser Permanente Washington Health Research Institute, Seattle, WA 98101, USA; 2 Vaccine and Infectious Disease Division; Fred Hutchinson Cancer Center, Seattle, WA 98109, USA; 3 GreenLight Biosciences, Medford, MA 02155, USA; 4 Department of Global Health; University of Washington, Seattle, WA 98105, USA; 5 Department of Biostatistics and Bioinformatics; Emory University, Atlanta, GA 30322, USA; 6 Public Health Sciences Division, Fred Hutchinson Cancer Center, Seattle, WA 98109, USA; 7 Department of Biostatistics; University of Washington, Seattle, WA 98195, USA

**Keywords:** Immunology, Immunological methods, Virology, Mathematical biosciences

## Abstract

Combination monoclonal broadly neutralizing antibody (bnAb) regimens are in clinical development for HIV prevention, necessitating additional knowledge of bnAb neutralization potency/breadth against circulating viruses. Williamson et al. (2021) described a software tool, Super LeArner Prediction of NAb Panels (SLAPNAP), with application to any HIV bnAb regimen with sufficient neutralization data against a set of viruses in the Los Alamos National Laboratory’s Compile, Neutralize, and Tally Nab Panels repository. SLAPNAP produces a proteomic antibody resistance (PAR) score for Env sequences based on predicted neutralization resistance and estimates variable importance of Env amino acid features. We apply SLAPNAP to compare HIV bnAb regimens undergoing clinical testing, finding improved power for downstream sieve analyses and increased precision for comparing neutralization potency/breadth of bnAb regimens due to the inclusion of PAR scores of Env sequences with much larger sample sizes available than for neutralization outcomes. SLAPNAP substantially improves bnAb regimen characterization, ranking, and down-selection.

## Highlights

• SLAPNAP predicts bnAb sensitivity of HIV-1 Env pseudoviruses using sequence data
• SLAPNAP increases precision for comparing bnAb regimen neutralization potential
• SLAPNAP also increases power for downstream sieve analysis

## Graphical abstract

**Figure:**
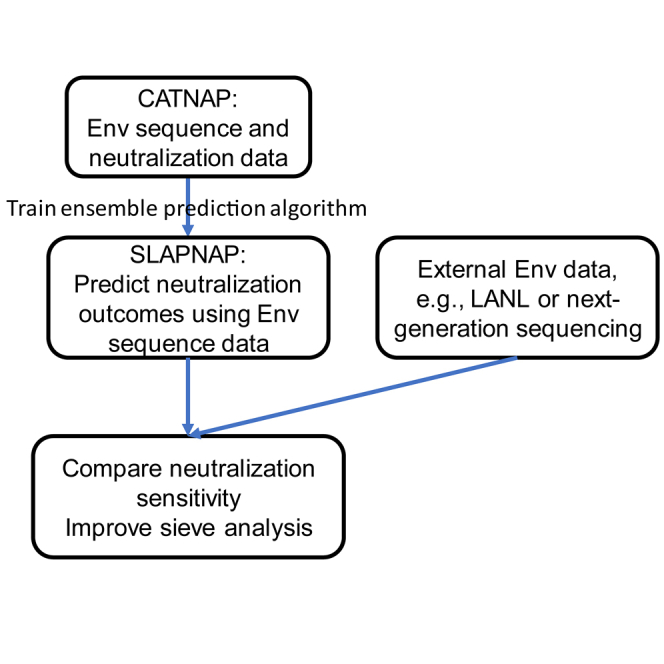

## Introduction

Extensive research has been conducted on prevention of HIV-1 through administration of monoclonal broadly neutralizing antibody (bnAb) regimens,^1^^,^^2^^,^^3^^,^^4^ with the antibody mediated prevention (AMP) randomized efficacy trials of VRC01 vs. placebo (HVTN 704/HPTN 085 and HVTN 703/HPTN 081, NCT02716675 and NCT02568215, respectively) providing proof of concept that a bnAb can prevent HIV-1 acquisition.^5^ While VRC01 did not prevent overall HIV-1 acquisition, prespecified analyses showed that estimated prevention efficacy of VRC01 (vs. placebo) against VRC01-susceptible strains [defined as 80% inhibitory concentration (IC_80_) < 1 μg/mL] was 75.4% (95% confidence interval, 45.5 to 88.9).^5^ The results of the AMP trials also advance knowledge toward a surrogate endpoint for HIV-1 acquisition: the predicted serum neutralization titer to an HIV-1 Env panel representing viruses to which a given study population is exposed to during follow-up.^6^ Once validated, such a surrogate endpoint would accelerate clinical testing and development of bnAb regimens and novel immunogens targeting bnAb induction, by providing a basis for regimen/immunogen characterization, comparison, and down-selection. The predicted serum neutralization titer surrogate endpoint also may provide a basis for provisional or traditional approval of bnAb regimens via clinical immunobridging (and, upon further scientific advances, perhaps also for provisional approval of bnAb-inducing vaccines).

With the aim of improving upon the results of the AMP trials in future efficacy trials, antibody engineering efforts have generated bnAb variants with increased potency and longer half-lives,^7^^,^^8^^,^^9^ which may enable administration about every 4 to 6 months at reasonable dose levels (similar to the dosing of medroxyprogesterone acetate, the widely used “birth control shot”^10^). Moreover, bnAb combinations targeting distinct Env epitopes have greater neutralization potency and breadth than their constituent single bnAbs, with *in vitro* neutralization coverage frequencies approaching 100%.^11^^,^^12^^,^^13^ Such combinations may also help reduce the possible evolutionary routes of HIV-1 escape.^14^ Thus, the HIV prevention field is currently prioritizing the movement of combination bnAb regimens or multispecific bnAbs toward efficacy trials^3^ (For simplicity, we use “bnAb regimen” hereafter to encompass single bnAbs, bnAb combinations, and multispecific bnAbs). Table S1 describes the pipeline of phase 1–2 trials underway or planned specifically through the HIV Vaccine Trials Network (HVTN) and the HIV Prevention Trials Network (HPTN) in collaboration with multiple partners, including the Vaccine Research Center (VRC), Beth Israel Deaconess Medical Center (BIDMC), International AIDS Vaccine Initiative (IAVI), The Rockefeller University, the Center for the AIDS Program of Research in South Africa (CAPRISA), the Aaron Diamond AIDS Research Center (ADARC), and the AIDS Clinical Trials Group (ACTG). In addition to the trials listed in Table S1, the DAIDS prevention clinical trials Networks and other groups are also preparing for efficacy trials of other bnAb regimens; Figure S1 summarizes the global pipeline of bnAb regimens.

In developing efficacious bnAb regimens, it is critical to understand the neutralization potency and breadth over time of a given regimen against HIV-1 Env panels that are representative of virus populations to which participants in future efficacy trials may be exposed.^12^^,^^15^ While in silico/*in vitro* analyses of bnAb potency and breadth inform projections of prevention potential of bnAb regimens, efficacy trial analyses explain *in vivo* impact on prevention efficacy.^16^ In particular, for any efficacy trial of a bnAb regimen (or multiple bnAb regimens), sieve analysis of viruses isolated from participants who acquire HIV-1 is conducted to assess how prevention efficacy of the regimen depends on features of exposing viruses, where the features are defined by immunological phenotypes such as level of neutralization resistance to the bnAb regimen (“neutralization sieve analysis”)^17^^,^^18^ and/or by amino acid (AA) sequence characteristics (“AA sequence sieve analysis”).

To enhance statistical power of the sieve analyses of the AMP trials, Magaret et al.^19^ modeled the sensitivity (susceptibility) of HIV-1 Env pseudoviruses to neutralization by the VRC01 clinical lot as a function of Env AA sequence features, based on data on 611 HIV-1 gp160 pseudoviruses from the Los Alamos National Laboratory (LANL) Compile, Analyze and Tally NAb Panels (CATNAP) database.^20^ A model estimated using the Super Learner^21^ predicted whether a given Env pseudovirus is resistant to VRC01 [defined by a right-censored 50% inhibitory concentration titer (IC_50_)]. This model provides a proteomic antibody resistance (PAR) score, defined as the predicted probability of VRC01 resistance or the predicted IC_50_ or IC_80_ neutralization readout for an Env gp160 sequence. The sieve analysis in AMP will study how VRC01 prevention efficacy varies with the PAR score of isolated acquired viruses. Moreover, Magaret et al.^19^ conducted variable importance analysis, finding that the most important AA sequence features for predicting VRC01 sensitivity (susceptibility) vs. resistance included 26 surface-accessible residues in the VRC01 and CD4 binding footprints, the lengths of gp120 and Env, the number of cysteines in gp120 and Env, and the presence or absence of four potential N-linked glycosylation sites. The hypothesis-driven sieve analysis in AMP focuses on these top-ranked features, which improves statistical power by reducing the extent of multiplicity adjustment that would be necessary for sieve analysis over a broader swath of AA sequence features. Bricault et al. conducted similar variable importance signature analyses as Magaret et al., except going beyond VRC01 to analyze all bnAbs across four antibody classes [CD4 binding site (CD4bs), V2, V3 glycan, and membrane proximal external region (MPER)] with sufficient data available in the LANL CATNAP database.^22^ Thus, Bricault et al. also provides a way to prioritize AA sequence features for sieve analysis in bnAb regimen efficacy trials.

Motivated by the bnAb pipeline discussed above, we extended Magaret et al. by developing a fully automated machine learning tool, Super LeArner Prediction of Nab Panels (SLAPNAP),^23^^,^^24^ for application to any combination bnAb regimen with sufficient neutralization outcome data available at LANL’s CATNAP database for a virus population of interest. Specifically, the needed neutralization outcome data are the *in vitro* IC_50_ or IC_80_ readouts for neutralization by each constituent bnAb in a combination regimen (or each parental bnAb of a multispecific bnAb) against a set of viruses/pseudoviruses. Whereas Williamson et al. described technical details of how to use the SLAPNAP tool to define PAR scores and estimate variable importance scores for any given bnAb regimen and set of viruses/pseudoviruses, this current companion article applies SLAPNAP to the bnAb regimens listed in Table S1 and describes its potential contributions to the characterization and comparison of HIV bnAb regimens, relevant for general bnAb researchers including clinicians and lab scientists.

## Results

### SLAPNAP tool calculates PAR scores and estimates variable importance for a given bnAb regimen and virus population

We briefly overview the SLAPNAP tool and illustrate its use through two examples. For a full description of the tool and initial validation results, including a comparison of prediction performance between SLAPNAP and the tools of Hake and Pfeifer^25^ and Rawi et al.,^26^ see Williamson et al.^24^ There, we observed no method dominated the others in all cases; since the Hake and Pfeifer^25^ and Rawi et al.^26^ methods do not directly incorporate combination bnAb regimens, we do not consider them further here.

SLAPNAP is a fully-automated, publicly available tool for training and evaluating machine learning models that predict *in vitro* neutralization susceptibility of Env pseudoviruses to a bnAb regimen based on Env gp160 AA sequence features. While previous studies, including Magaret et al. and Williamson et al., have used the term “sensitive” to describe Env pseudoviruses that may be neutralized by a given bnAb, in the remainder of this manuscript we will use the term “susceptible” to avoid possible confusion with the sensitivity measure of binary classification performance. Once a bnAb regimen and neutralization outcome(s) of interest (e.g., IC_80_ or IC_80_ < 1 μg/mL) have been identified, a cross-validated ensemble prediction algorithm is built using AA sequence features to predict the outcome(s) of interest. Variable importance – both algorithm-specific variable importance and general variable importance evaluated by metrics independent of the specific machine learning algorithms (so-called “intrinsic variable importance”) – can also be computed. By default, a large library of candidate prediction algorithms is included in the ensemble, but this library can be edited by the user. See the “STAR Methods” section for further details.

We analyzed two example combination bnAb regimens: VRC07-523-LS + PGT121 and VRC07-523-LS + PGT121 + PGDM1400. Both combination regimens are currently being studied in HVTN 130/HPTN 089 (Table S1). The neutralization outcome of interest for both regimens was the binary indicator that combination IC_80_ < 1 μg/mL (“susceptible” vs. “resistant”), where combination IC_80_ was determined using the individual-bnAb IC_80_ values and the additive model of Wagh et al.^12^ We chose this outcome based on the finding from the AMP trials that VRC01 prevented acquisition of the subset of circulating HIV-1 strains with an IC_80_ < 1 μg/mL.^5^ The choice of additive combination model has been validated both by Wagh et al.^12^ and Williamson et al.^24^ In addition, the HVTN 130/HPTN 098 study infused study volunteers with the 3 monoclonal antibodies PGDM1400 + PGT121 + VRC07-523LS and studied observed serum ID_80_ titers vs. predicted combination ID_80_ titer against a global panel of 12 HIV-1 viruses based on the Bliss-Hill model. Results were highly concordant (Figure 6 in Sobieszczyk et al., conditionally accepted at Lancet HIV). The full SLAPNAP specification (including candidate prediction algorithms and other arguments) for both bnAb regimens is provided on GitHub (https://github.com/bdwilliamson/clinical_slapnap). For each regimen, we assessed the cross-validated area under the receiver operating characteristic curve (CV-AUC) of the resulting PAR score for predicting the combination IC_80_, the intrinsic variable importance of both individual AA sites and groups of AA sites (defined in Methods), and the predictive importance of residues within the top-performing individual algorithm from the Super Learner library. The analysis used all Env gp160 sequences at LANL with corresponding neutralization IC_80_ data at CATNAP (as given in Table S1). The prediction performance of the remaining bnAb regimens from Table S1 is provided in Table 1, along with the estimated performance for predicting the “multiple susceptibility” outcome that IC_80_ < 1 μg/mL for at least one bnAb in the combination (if applicable). Additional performance metrics, including classification accuracy, sensitivity, specificity, positive and negative predictive value, and Matthews correlation coefficient, are provided in Tables S2 and S3. Care must be taken when evaluating the results for predicting binary outcomes (susceptibility and multiple susceptibility), because the effective sample size (i.e., the number of observations in the minority class) may be small. This could contribute to observed CV-AUCs that are closer to 0.5 for a combination regimen than for the constituent bnAbs.

**Table 1:** SLAPNAP calculation of prediction accuracy of the proteomic antibody resistance (PAR) scores of gp160 sequences for predicting HIV-1 pseudovirus susceptibility to each bnAb regimen in Table S1

bnAb regimen	CV-AUC [95% CI]		CV-R^2^ [95% CI]
	Susceptibility	Multiple susceptibility	IC_80_
VRC01	0.744 [0.641, 0.825]	–	0.345 [0.266, 0.416]
VRC07-523-LS	0.728 [0.581, 0.837]	–	0.193 [0.072, 0.298]
PGT121	0.850 [0.765, 0.908]	–	0.571 [0.473, 0.65]
VRC26.25	0.867 [0.769, 0.928]	–	0.53 [0.442, 0.604]
PGDM1400	0.873 [0.792, 0.925]	–	0.501 [0.398, 0.586]
VRC07-523-LS + PGT121	0.768 [0.596, 0.882]	0.781 [0.623, 0.885]	0.316 [0.219, 0.402]
VRC07-523-LS + VRC26.25	0.689 [0.50, 0.831]	0.749 [0.56, 0.875]	0.373 [0.295, 0.443]
VRC07-523-LS + PGDM1400	0.638 [0.454, 0.789]	0.669 [0.482, 0.815]	0.255 [0.198, 0.308]
VRC07-523-LS + 10-1074	0.783 [0.564, 0.909]	0.784 [0.619, 0.891]	0.319 [0.215, 0.409]
VRC07-523-LS + PGT121 + PGDM1400	0.730 [0.499, 0.879]	0.708 [0.454, 0.876]	0.181 [0.147, 0.214]
VRC01/PGDM1400/10e8v4	0.810 [0.583, 0.929]	–	0.254 [0.083, 0.392]

Susceptibility is defined differently for single bnAbs and combination regimens. For single bnAbs, susceptibility is defined as IC_80_ < 1 μg/mL. For a combination regimen with J bnAbs, susceptibility is defined as combination IC_80_ < 1 μg/mL, where combination IC_80_ = ${\left(\sum_{j=1}^{J}{IC}_{80,j}^{-1}\right)}^{-1}$. Multiple susceptibility is defined as IC_80_ < 1 μg/mL for at least one bnAb in a combination regimen. For predicting susceptibility and multiple susceptibility, point and 95% confidence interval (CI) estimates of cross-validated area under the receiver operating characteristic curve (CV-AUC) are used; for predicting IC_80_, point and 95% CI estimates of CV-R^2^ are used. See also Figures S1, S2, Tables S1–S3, and S4.

We first discuss the predictive performance of SLAPNAP for VRC07-523-LS + PGT121 and VRC07-523-LS + PGT121 + PGDM1400 (Table 1). The CV-AUC for classifying combination IC_80_ above or below 1 μg/mL was 0.730 (95% confidence interval (CI) of [0.499, 0.879]) for the three-bnAb regimen and 0.768 [0.596, 0.882] for the two-bnAb regimen. Since a CV-AUC of 0.5 indicates null predictive performance, the CV-AUCs presented above indicate moderate predictive ability. The slightly lower CV-AUC for the three-bnAb regimen may be due in part to a larger observed imbalance between susceptible and resistant pseudoviruses: 374 pseudoviruses were susceptible to the three-bnAb combination, while only 26 were resistant [susceptible (resistant) defined by combination IC_80_ < (≥) 1 μg/mL]; 347 pseudoviruses were susceptible to the two-bnAb combination, while 53 were resistant. We compared the predictive performance of these two combination-bnAb regimens with the single bnAbs VRC07-523-LS, PGT121, and PGDM1400. The CV-AUCs of the resulting PAR scores were 0.728 [0.581, 0.837], 0.850 [0.765, 0.908], and 0.873 [0.792, 0.925], for VRC07-523-LS, PGT121, and PGDM1400, respectively, with 315 pseudoviruses classified as susceptible and 85 classified as resistant to VRC07-523-LS, 253 classified as susceptible and 289 classified as resistant to PGT121, and 258 classified as susceptible and 263 classified as resistant to PGDM1400.

We observed similar patterns of variable importance for both bnAb combinations. The gp120 CD4 binding sites had the largest estimated intrinsic variable importance in both combination regimens; the p value from a hypothesis test of zero intrinsic importance for this group was less than 0.05 for both regimens. The gp120 V3 region also ranked highly in the VRC07-523-LS + PGT121 regimen, while the gp120 V2 region also ranked highly in the VRC07-523-LS + PGT121 + PGDM1400 regimen. Individual AA sites and geometric features estimated to be important across both regimens included sites 348 and 471 (all sites are referenced to HXB2) and the length of V2. Several AA sites also were estimated to be important both intrinsically and based on the best-performing algorithm within the Super Learner (for both regimens, a random forest). For the three-bnAb regimen, sites 142 and 602 ranked in the top 20 most important variables using either definition of variable importance. For the two-bnAb regimen, sites 337, 365, and 371 ranked in the top 20 using either definition of variable importance. While the variables identified by these two types of importance are not expected to agree exactly, some overlap suggests a well-calibrated approach, and the sites identified using this approach may be of interest in future studies. The full set of results, including variable importance ranks, is available on GitHub.

Prediction performance for all bnAb regimens was mixed (Table 1; Tables S2, S3, S4). CV-AUC for prediction of susceptibility ranged from 0.638 (for VRC07-523-LS + PGDM1400) to 0.873 (for PGDM1400); CV-R^2^ for prediction of IC_80_ ranged from 0.181 (VRC07-523-LS + PGT121 + PGDM1400) to 0.571 (PGT121). The maximum F1 score achieved for each bnAb regimen when predicting susceptibility or multiple susceptibility ranged from 0.63 (VRC01) to 0.962 (VRC07-523-LS + PGT121 + PGDM1400), while the maximum Matthews correlation coefficient ranged from 0.11 (VRC07-523-LS + PGDM1400) to 0.59 (VRC26.5).

### SLAPNAP improves characterization, ranking, and down-selection of bnAb regimens

In this section, we first discuss a predicted neutralization titer-based study endpoint for phase 1–2 bnAb trials that may be useful for comparing bnAb regimens; we refer to this as the “Prevention Efficacy Potential” (PE-potential) endpoint. A key element of the PE-potential endpoint is the distribution of IC_80_ values of a population of viruses to which participants in a future efficacy trial may be exposed. Accordingly, for a given bnAb regimen, it is of interest to estimate the geometric mean of the combination IC_80_ endpoint for various populations of circulating viruses that circulate in different geographic regions. We describe how the SLAPNAP tool improves statistical precision for comparing the geometric mean combination IC_80_ across bnAb regimens for various populations of circulating viruses that are estimated to circulate in certain geographic regions.

#### PE-potential endpoint

Potential HIV prevention bnAb regimen(s) must be ranked and down-selected, as only a small number of large-scale HIV prevention efficacy trials are feasible in the immediate time horizon. Building on Gilbert et al.,^6^ Figure S2 summarizes the PE-potential endpoint. If exposures to HIV-1 in the future efficacy trial occur approximately uniformly over follow-up, then the PE-potential endpoint approximately captures a challenge-trial endpoint of day-of-challenge neutralization titer against the challenge virus, averaged over expected exposures/challenges. Other factors for ranking and down-selecting bnAb regimens include safety, cost, schedule, manufacturability, and non-neutralizing immunological effector functions. Some of these factors can be integrated into variants of the PE-potential endpoint, e.g., by scaling the endpoint by the total mass of bnAb delivered during the time period. Research on elements a−d from Figure S2 has included building bnAb pharmacokinetic (PK) models^27^^,^^28^^,^^29^^,^^30^; conducting statistical/machine learning of pseudovirus sequence predictors of neutralization susceptibility to mAbs^19^^,^^22^; and using Bliss-Hill models to combine individual IC_80_ readouts across multiple bnAbs,^12^^,^^15^^,^^31^ demonstrating that experimental serum neutralization titers can be well predicted from an integration of the separate data elements a−d (Figure S2).^6^^,^^32^ The PE-potential endpoint integrates data from multiple sources including population PK/pharmacodynamic (PD) modeling of bnAb serum concentrations and neutralization, databases on virus susceptibility to bnAb neutralization, and molecular epidemiology of viruses circulating in geographic regions where future prevention efficacy trials may occur.

#### Improved comparisons of bnAb regimens by SLAPNAP

We illustrate how the use of SLAPNAP can improve the comparison of bnAb regimens when there are Env sequence datasets available from populations of interest (outside of CATNAP) for which data are not available on neutralization susceptibility to the bnAb regimens of interest. We use two metrics for comparing bnAb regimens: the estimated geometric mean IC_80_ and the estimated probability that IC_80_ < 1 μg/mL, where the estimates are computed based on all viruses with IC_80_ values (“Reference approach”) or based on all viruses including those that have Env-sequence PAR scores but no IC_80_ values (“SLAPNAP-augmented approach”). For each bnAb regimen and each outcome type (quantitative vs. binary), we generated neutralization outcome and PAR score data compatible with the observed predictiveness of SLAPNAP (Table 1), ensuring that the resulting PAR scores have some predictive utility. We varied the percentage increase in the total number of viruses available for analysis when using the SLAPNAP-augmented approach compared to the Reference approach (see the “quantification and statistical analysis” section for more details). With these simulated data, we estimated each mean outcome value and the relative efficiency of the SLAPNAP-augmented approach vs. the Reference approach using the methods described in “Statistical Analyses”. As a comparison, we included the estimates from an “oracle approach” that had access to IC_80_ neutralization values for all viruses; this estimator would not be available in practice.

**Figure 1:**
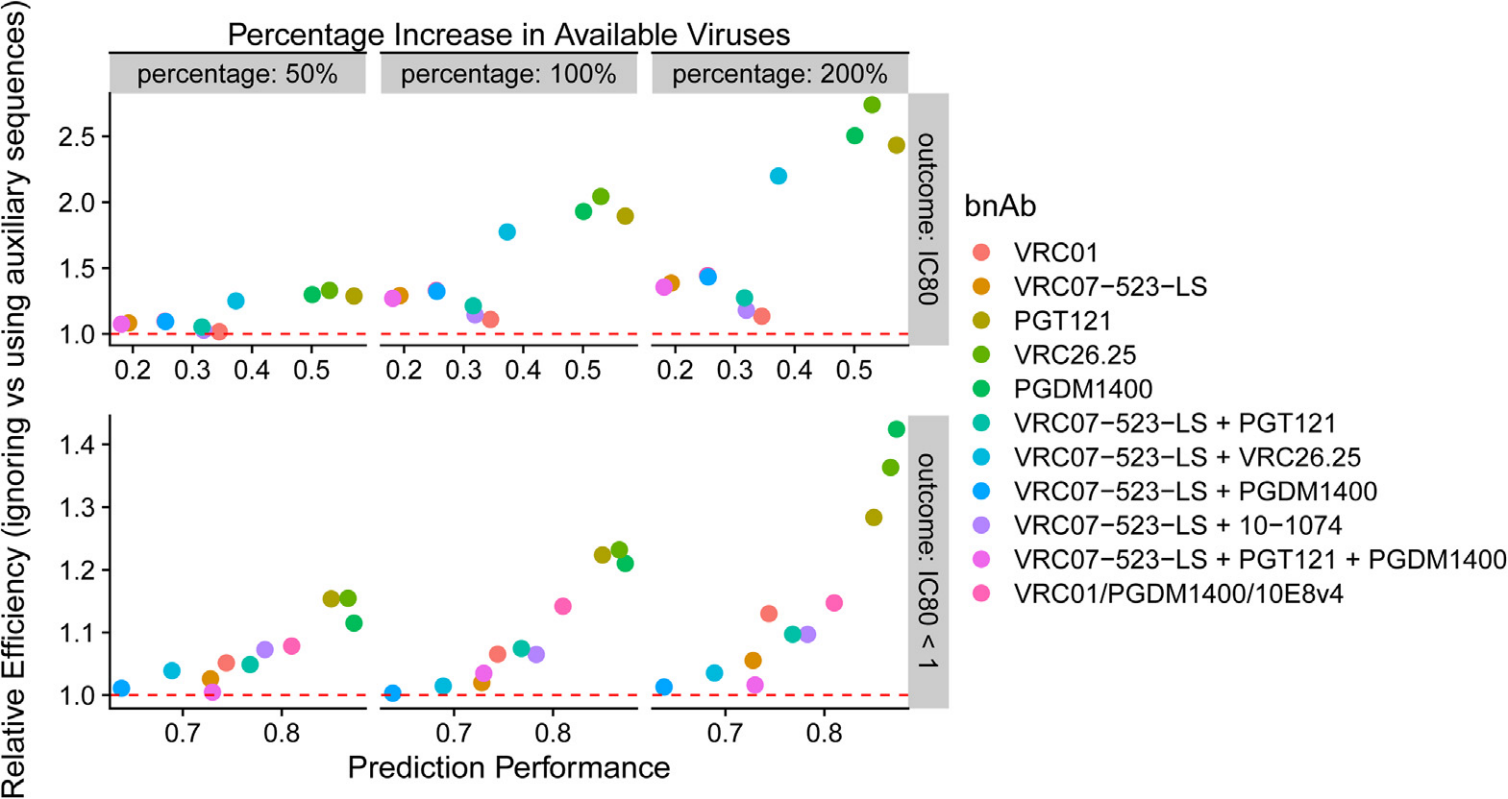
Relative efficiency (ratio of the sample variances of the estimated mean outcome value for the Reference approach vs. SLAPNAP-augmented approach, taken over 1000 simulated replicates) versus estimated SLAPNAP-prediction performance for each bnAb regimen listed in Table S1 Top row: Prediction of IC_80_. Bottom row: Prediction of binary IC_80_ < 1 μg/mL. Columns denote the percentage increase in the number of viruses included in the SLAPNAP-augmented approach when adding viruses with data on Env sequence data only to viruses with data on both Env sequence and IC_80_. The bnAb regimens are differentiated by color; each point represents the ratio of the Monte-Carlo variances taken over 1000 simulated replicates. See also Figures S3–S8.

We present the results for the simulated data in Figure 1. Each point in the figure is a ratio of the Monte-Carlo sample variances (Reference/SLAPNAP-augmented) of the estimated mean neutralization outcome, taken over 1000 simulated replicates. For both neutralization outcomes, the bnAb regimens with the highest estimated predictiveness of the SLAPNAP algorithm also have the largest gain in efficiency from including the viruses with only Env sequences and no measured IC_80_. Both approaches are unbiased (Figure S3) as suggested by theory,^33^ with magnitude of bias (mean difference between the estimated and true neutralization outcome values over 1000 simulated replicates) less than 0.003 in all cases, and relative percent bias [mean over 1000 simulated replicates of {(estimated neutralization value – true neutralization value)/true neutralization value} x 100%] less than 3% in all cases (Figure S4). In all scenarios studied, the relative efficiency exceeds one, indicating a consistent advantage to including Env sequences over using measured IC_80_ alone. While the oracle estimator was more efficient than both the reference and SLAPNAP-augmented estimators, the SLAPNAP-augmented estimator had a smaller loss of efficiency than the reference estimator (Figures S5 and S6). A sensitivity analysis showed that these results are robust to our initial choice of data-generating mechanism (Figures S7 and S8).

For a given dataset it is possible that an estimator that uses inverse probability weights to include Env sequences (as we did here) could provide worse performance than using IC_80_ alone; for example, if some of these weights have outlying values. We suggest that metrics measuring how well the PAR score predicts log IC_80_ can be used as a guide for whether Env sequence data with no corresponding IC_80_ data can be reliably included. Specifically, if CV-R^2^ exceeds 0.2, then the augmented estimator we used in this simulation is expected to be approximately 20% more efficient than the estimator ignoring the extra Env sequences, providing a substantial improvement (see, e.g., Figure 2 in Gilbert et al; ^34^). We recommend that external Env sequences be included if the external Env sequence data contribute at least 50% more observations than the measured IC_80_ alone and if CV-R^2^ is at least 0.2.

**Figure 2:**
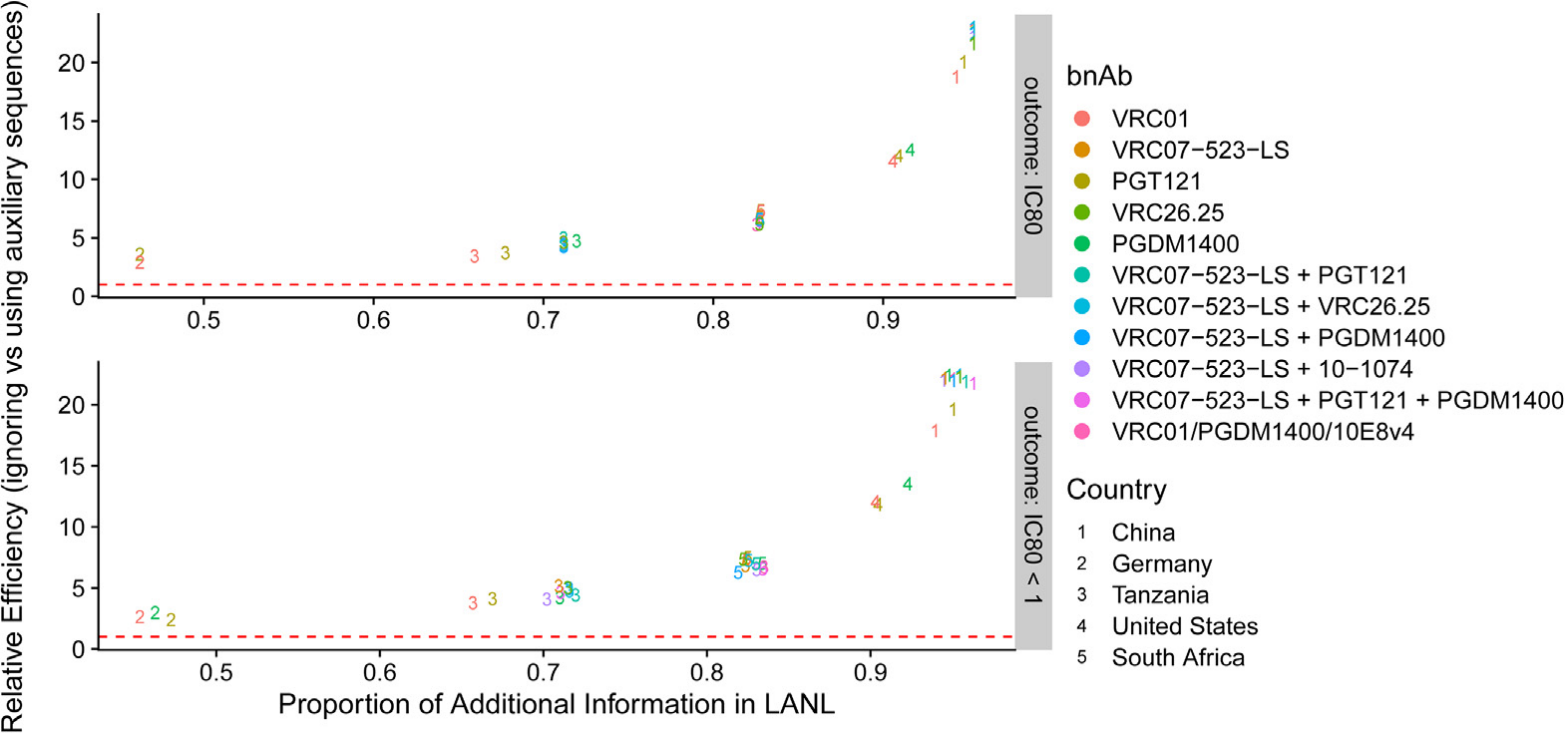
Bounded relative efficiency (ratio of the squared widths of the 95% percentile bootstrap confidence interval for the mean neutralization outcome for the Reference approach vs. SLAPNAP-augmented approach bounded above by the maximum possible efficiency for the given combination of bnAb regimen and country, $\frac{\#\left\{sequences\;available\;in\;both\;LANL\;and\;CATNAP\right\}}{\#\left\{sequences\;available\;in\;CATNAP\right\}}$) versus the proportion of additional numbers of Env sequences in LANL compared to CATNAP Top row: Prediction of IC_80_. Values were imputed in SLAPNAP at two times the right censoring value reported in CATNAP, as in ref. 19 Bottom row: Prediction of binary IC_80_ < 1 μg/mL. Columns show the estimated prediction performance for each bnAb regimen listed in Table S1. The bnAb regimens are differentiated by color, while countries are differentiated by the plotting symbol (1–8). Only country/bnAb regimen combinations with at least 30 pseudoviruses in CATNAP were analyzed. Clades represented overall: 01_AE, 02_AG, 07_BC, A1, B, C, D, Other. Clades by country in the figure: China: 01_AE, 02_AG, 07_BC, B, C, Other. (majority: Other, 01_AE, B); Germany: 02_AG, B, C, Other. (majority: B); Tanzania: A1, C, D, Other. (majority: C, Other); United States: 01_AE, 02_AG, A1, B, C, D, Other. (majority: B); South Africa: A1, B, C, D, Other (majority: C).

For planning prevention efficacy trials, it is useful to evaluate bnAb regimen potential prevention efficacy by global region (e.g., for individual countries or sets of countries). Some countries have far more Env sequences available without than with measured neutralization outcomes. To estimate the impact of applying SLAPNAP for country-specific analyses, for each bnAb regimen listed in Table S1 we considered all countries with more than 30 IC_80_ values in CATNAP: China, Germany, Kenya, Malawi, South Africa, Tanzania, Uganda, and the US. Next, we matched these neutralization outcomes to all available Env sequences in the LANL HIV sequence database for each country. Then, for each bnAb regimen and each country, we used SLAPNAP to generate PAR scores for each Env sequence, and considered estimation of the same two mean outcomes studied for Figure 1, and computed relative efficiency using 95% percentile bootstrap confidence intervals for the mean outcomes. We present the results of this analysis in Figure 2. The results follow a similar pattern to those observed in Figure 1, with inclusion of greater amounts of auxiliary sequences/PAR scores improving relative efficiency.

### SLAPNAP improves sieve analysis in bnAb prevention efficacy trials

Sieve analysis, for example as applied in refs.,^35^^,^^36^^,^^37^^,^^38^ conducts both (1) hypothesis-driven analyses of selected AA sequence features, with the features selected by immunology, virology, and structural biology data; and (2) unbiased analyses including all Env AA positions exhibiting enough variability to make it possible for a statistical test to yield a significant p value for a difference between virus genotypes acquired in the bnAb group vs. placebo group. The former analyses typically have greater power because of the attenuated type I error (multiple hypothesis testing) adjustment and because the incorporation of biological data may inform the identification of more biologically plausible, likely sieve effects, while the latter analyses can generate hypotheses about relevant AA sequence features.

We illustrate how the use of SLAPNAP can improve the power of sieve analyses in prevention efficacy trials. We simulated data that mimic the AMP trial data under the hypothesis that one CD4 binding site AA identified as important for predicting VRC01 neutralization susceptibility in the Statistical Analysis Plan for the AMP sieve analysis^39^ yields truly different prevention efficacy that varies by the AA residue at the specified position (AA residue D at position 230; see the “quantification and statistical analysis” section for more details). We then consider two sieve analyses of these simulated data. First, we consider AA site-scanning sieve analysis; this type of sieve analysis has so far been done for all six HIV vaccine efficacy trials from 2003-2017.^35^^,^^36^^,^^37^^,^^38^^,^^40^^,^^41^^,^^42^ In this analysis, we apply a Lunn and McNeil test^43^ for differential prevention efficacy at each AA site in gp120 that passed a minimum variability filter in the AMP data (a total of 414 sites), where detection is defined by the resulting Holm-Bonferroni adjusted two-sided p value from this test being less than 0.05. We compute the power of this procedure as the Monte-Carlo average of true detections over 1000 replicates of the simulation. Next, we consider a “priority” sieve analysis restricted to the 15 pre-identified sites from AMP,^39^ and again use the Lunn and McNeil test with multiplicity-adjusted p value threshold of 0.05. We compute the power of this procedure identically as above.

**Figure 3:**
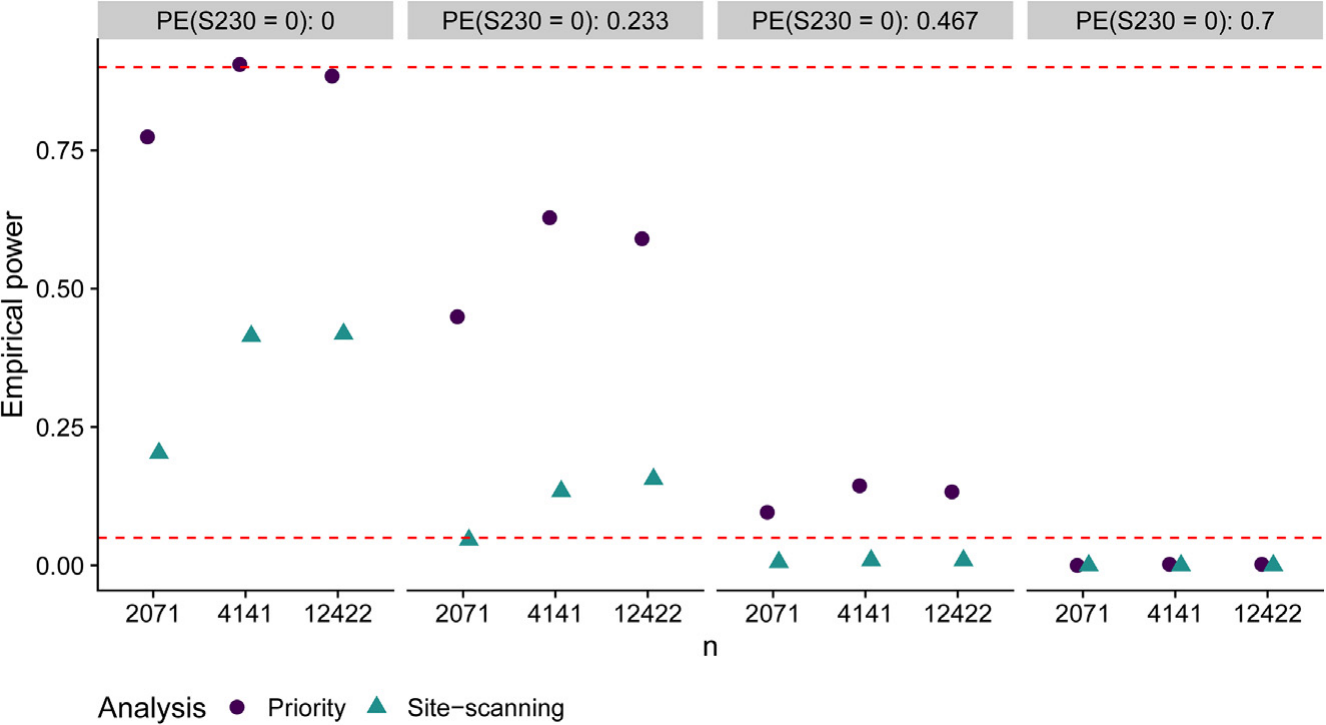
Empirical power of sieve analyses under different sieve alternative hypotheses, ranging from a large sieve effect (left-column) to the null hypothesis of no sieve effect (right-column) (A “sieve effect” is differential prevention efficacy against S230 HIV-1 vs. against not S230 HIV-1.) Power of a Lunn and McNeil^43^ test for detecting a sieve effect is displayed for an unbiased site-scanning analysis over 414 amino acid (AA) positions in gp120 that passed a minimum variability filter in AMP, and for a priority hypothesis-driven analysis that restricted to the 15 AA positions in gp120 pre-identified as important by SLAPNAP.

We present the results of this analysis in Figure 3. Under the largest sieve effect sizes (the left two columns of Figure 3), there is a substantial increase in power when prioritizing 15 sites instead of including all 414 sites, with power near 90% for detecting a sieve effect in the priority analysis. The priority analysis results in uniformly higher power than the complete site-scanning analysis, where the increase in power decreases as the magnitude of the sieve effect decreases. Both analyses control type I error below the nominal 0.05 level under the sieve null hypothesis of non-differential vaccine efficacy (the rightmost column of Figure 3).

## Discussion

This companion article to Williamson et al.^24^ describes applications of the SLAPNAP neutralization prediction tool to combination monoclonal bnAb HIV prevention research. For a CATNAP dataset on neutralization susceptibility phenotypes (e.g., IC_80_) of a combination HIV bnAb regimen against a panel of HIV pseudoviruses, plus the Env sequences for all pseudoviruses in the panel, SLAPNAP builds models for predicting neutralization susceptibility from Env sequences and provides tools for quantifying and visualizing results, including PAR scores and estimates of variable importance scores. We illustrated two specific applications of SLAPNAP, the first demonstrating that it can be used to increase precision for comparing neutralization susceptibility among several combination bnAb regimens undergoing evaluation in phase 1 trials by including Env-sequence predictions of neutralization susceptibility in the analysis. For geographic regions and time periods for which limited numbers of pseudoviruses with measured neutralization outcomes are available, SLAPNAP can greatly improve precision. The second application demonstrated that SLAPNAP can improve hypothesis-driven sieve analysis by specifying putatively neutralization-relevant Env sequence features. This application applies to HIV-1 vaccine efficacy trials as well as to HIV-1 monoclonal bnAb prevention efficacy trials, since sequence features impacting passive and active immunization are likely to overlap given that similar immunologic mechanisms (e.g., antibody-mediated neutralization of virus) likely contribute to HIV-1 prevention through passive and/or active immunization.

The current implementation of SLAPNAP focuses on the neutralization readouts IC_50_, IC_80_, and instantaneous inhibitory potential (IIP),^44^ because IC_50_ and IC_80_ are available in CATNAP, and IIP can be calculated from the IC_50_ and IC_80_. Other metrics based on other features of the neutralization curve such as maximum percent inhibition (MPI) and complete neutralization (defined by MPI ≥ 95% as in ref. 12,15 or more stringently as MPI = 100%) may also be important, because it is possible that bnAb protection would be abrogated by minority variants in an exposing virus swarm; in such a case, complete neutralization could be hypothesized to correlate more strongly with *in vivo* prevention efficacy to block HIV-1 acquisition than other neutralization readouts. Moreover, bnAb regimens may rank differently based on MPI and complete neutralization than on the other metrics. Currently there do not exist public databases with MPI and complete neutralization data; such databases are needed.

SLAPNAP has applications to next-generation sequencing data. While the HVTN’s previous vaccine efficacy trials used single genome-amplification sequencing, which yields about 5–20 HIV sequences per participant, the AMP trials were the first HVTN studies to use long-read PacBio technology, which typically yields 200 HIV sequences per participant. The greater PacBio sequencing depth increases potential insights from sieve analysis by resolving rare variants to a higher precision. SLAPNAP is valuable for such deep sequencing data by providing estimated neutralization susceptibility values (PAR scores) for all sequence reads in a given participant sample. In contrast, it would be extremely laborious and costly to directly measure IC_80_ values of all individual sequence reads given the need to make pseudoviruses for each read; for example, more than 30,000 individual Env sequence reads were measured in the AMP trials.

Newer HIV monoclonal antibodies recently entering clinical studies include 3BNC117-LS-J and VRC07-523-LS (targeting the CD4 binding site), 10-1074-LS-J and PGT121LS (targeting V2), and CAP256V2LS^45^ and PGDM1400LS (targeting the V3 glycan). Next-generation enhanced monoclonal antibodies include VRC01.23LS^9^ as well as enhanced versions of PGT121 and PGDM1400 (e.g., ePGT121.v2-LS, ePGDM1400v9-LS). While it is of considerable interest to compare neutralization susceptibility profiles of combination bnAb regimens of these antibodies, CATNAP data are not yet available to support these analyses (as of October 17, 2022). As CATNAP acquires neutralization susceptibility data for these antibodies, SLAPNAP will be poised for rapid contributions given the large number of available Env sequences.

### Limitations of the study

CATNAP and SLAPNAP each have limitations. CATNAP has been critiqued for containing many older viruses, with concern that their neutralization susceptibility profiles may not well represent those for contemporary circulating viruses, especially an issue if frequencies of resistant viruses increase over time,^46^ as may occur with antigenic drift. SLAPNAP may help address this issue as it is easier to curate and maintain contemporary Env sequence databases than neutralization phenotype databases, due to the large additional effort needed to construct pseudoviruses and experimentally measure IC_80_ values. Thus, SLAPNAP can more efficiently and feasibly contribute neutralization information on contemporary circulating viruses over time. Yet it is still a limitation, because the genetic predictor PAR scores are built based on the number of viruses in CATNAP with neutralization measured against the bnAb regimen of interest.

Another critique of CATNAP is that much of its neutralization data were measured for pseudoviruses produced in 293T cells, with concern about biological relevance compared to alternative neutralization measurements (e.g., IC_80_ values measured against contemporaneous clinical isolates, including but not limited to those produced in PBMCs,^47^). One response is that the AMP trials^5^^,^^6^ and non-human primate challenge trials^48^ have shown the pseudovirus TZM-bl target cell assay to perform well as a statistical corelate of protection, such that even in the case that the assay does not capture a mechanistic correlate of protection, it is nonetheless supported to be suited for use as a surrogate endpoint for predicting prevention efficacy and hence with utility as a biomarker for ranking and down-selection of bnAb regimens. Vaccine research has a long history of accepting non-mechanistic correlates of protection as surrogate endpoints for vaccine decision-making (e.g., binding antibody assays) even though they do not completely measure the functional mechanism of protection.^49^

## STAR★Methods

### Key resources table

**Table:** 

REAGENT or RESOURCE	SOURCE	IDENTIFIER
**Software and algorithms**		

slapnap: Super LeArner Prediction of NAb Panels	Williamson et al.^24^	https://benkeser.github.io/slapnap
Original code for compiling the CATNAP data	This paper	https://doi.org/10.5281/zenodo.6983658
Simulation plan for generating additional data used in “Improved Comparisons of bnAb regimens by SLAPNAP” and in “SLAPNAP improves sieve analysis in bnAb efficacy trials”	This paper	Supplemental information of this paper
Full SLAPNAP specification (including candidate prediction algorithms and other arguments) for the two combination bnAb regimens VRC07-523-LS + PGT121 and VRC07-523-LS + PGT121 + PGDM1400	This paper	https://github.com/bdwilliamson/clinical_slapnap

### Resource availability

#### Lead contact

Further information and requests for resources should be directed to and will be fulfilled by the Lead Contact, Peter B. Gilbert (pgilbert@fredhutch.org).

#### Materials availability

This study did not generate new unique reagents.

### Method details

While the SLAPNAP tool is described in full on GitHub (https://benkeser.github.io/slapnap) and in Williamson et al.,^24^ we provide a brief description of the tool here.

Once a bnAb regimen and neutralization outcome of interest have been identified, SLAPNAP uses the Super Learner^21^ to predict the outcome. The Super Learner is a cross-validated ensemble of individual algorithms; cross-validation is used to determine the convex combination of the individual algorithms that results in the lowest cross-validated risk. Based on a fitted Super Learner model, SLAPNAP uses cross-validated area under the receiver operating characteristic curve (CV-AUC) or cross-validated R-squared (CV-R^2^), as appropriate for the given outcome type, to measure the prediction performance of the measured AA sequence features. Confidence intervals for CV-AUC and CV-R^2^ based on the efficient influence function are also provided.

The candidate algorithms in the Super Learner and the number of cross-validation folds may need to be tailored based on the analysis at hand, and can be based on, for example, the sample size (or effective sample size in binary-outcome settings, equal to the number in the minority class) (see, e.g., ref. 51). We have used several such approaches in other settings, including applying a screen to all individual learners to allow only a maximum of p input features for any given model, where p scales with the number of observations.^52^ In general, five- or ten-fold cross-validation has been shown to yield good performance results.^53^ In some small-sample settings (or binary outcome settings with number of events less than, e.g., 50), leave-one-out cross-validation can be used instead, and in this situation we recommend using only non-adaptive learners (e.g., generalized linear models and lasso).^52^

To investigate the amount of data sufficient for using SLAPNAP, we subsampled from the CATNAP data for VRC01, as described in “Quantification and Statistical Analyses”. For each sample size n∈{10,20,…,200}, we (i) took a sample of size n; and (ii) computed the cross-validated mean-squared error (CV-MSE) using lasso to predict log_10_ IC_80_. We repeated steps (i) and (ii) 2500 times for each n. In Figure S4, we display the results of this experiment. CV-MSE is fairly small for all sample sizes, but the largest jumps are between n = 10 and n = 20 and n = 20 and n = 30, suggesting that a sample size of 30 could be a reasonable lower bound for training SLAPNAP.

Variable importance estimates can also be computed. Two approaches to variable importance estimation are available in SLAPNAP. The first is based on population prediction performance, also referred to as *intrinsic* importance.^54^^,^^55^ In this approach, variable importance is defined as the increase in population prediction performance when the features of interest are added to a set of adjustment features (either all remaining features or the geographic potential confounding variables; see Equation 2 in Williamson et al.^55^). This population prediction performance can be measured by nonparametric R^2^ for continuous outcomes and nonparametric AUC for binary outcomes. Both 95% confidence interval estimates and p-values based on a test of the zero-importance null hypothesis are returned by SLAPNAP. The second definition of variable importance is based on the fitted learner/algorithm, also referred to as *predictive* or *extrinsic* importance. Examples of extrinsic importance measures are provided in Section 2.3 of Williamson et al.^24^

### Quantification and statistical analysis

#### S1 Introduction

The analyses address the following goals:

1. *(Simulation)* Estimate the number of pseudoviruses with neutralization data available that is sufficient to use SLAPNAP;
2. (*Data analysis*) Obtain PAR scores and estimate predictive performance of SLAPNAP^24^ for several bnAb regimens;
3. (*Simulation*) Determine if using external data (i.e., Env sequences) results in variance reduction for estimating the mean log_10_ IC_80_ value and/or the probability that IC_80_ < 1 μg/ml;
4. (*Data analysis*) Determine if using Env sequences from LANL results in variance reduction for estimating the mean log_10_ IC_80_ value and/or the probability that IC_80_ < 1 μg/ml for several bnAb regimens in several countries, compared to using data from the Compile, Analyze and Tally NAb Panels (CATNAP) database^20^ alone; and,
5. (*Simulation*) Determine if using SLAPNAP can improve sieve analysis.

In the next sections, we describe our proposed approach to answering these questions. All analysis code is available on GitHub^56^ and at Zenodo.^50^

#### S2 Simulation: Number of pseudoviruses needed to use SLAPNAP

In this section, we provide some guidance on the number of pseudoviruses with measured neutralization outcomes necessary to run SLAPNAP.

We proceed as follows: for each sample size n∈{10,20,…,200}, we

1. Sample a dataset of size n from the CATNAP data for VRC01 (dataset from Section S3);
2. Compute the 5-fold cross-validated mean-squared error (CV-MSE) using a lasso regression model (with 5-fold cross-validation to select the tuning parameter) to predict log_10_ IC_80_.

We repeated this process 2500 times for each sample size, and computed the average CV-MSE over these 2500 replications. The results are presented in Figure S9.

#### S3 Data analysis: SLAPNAP tool gives PAR scores and estimates of variable importance for a given bnAB regimen and virus proportion

For this analysis, we ran SLAPNAP^24^ for each bnAb regimen provided in Table 1, providing both a predictor of the mean log_10_ IC_80_ value and a predicted probability that IC_80_ < 1 μg/ml for each bnAb regimen. Because values in CATNAP can be reported as right-censored, in SLAPNAP we impute these values at two times the right-censoring value, as in Magaret et al.^19^ We used a Super Learner ensemble consisting of random forests,^57^ gradient boosted trees,^58^ and the elastic net,^59^ each with varying tuning parameters; these candidate learners are described more fully in Williamson et al.^24^ In some cases, it may improve prediction performance (and reduce computation time) to remove binary AA features with fewer than *x* values in the minority class; this is also referred to as a minimum variability filter. For example, Magaret et al.^19^ excluded binary AA features with fewer than 3 values in the minority class (i.e., the minimum variability threshold was 3). Here, we considered minimum-variability thresholds of zero and four for the AA features. The PAR scores for each bnAb regimen are the predictions from the final Super Learner ensemble for predicting the outcome of interest (IC_80_ < 1 μg/ml or quantitative log_10_ IC_80_); the final ensemble is a convex combination of the individual candidate learners that minimized the cross-validated risk (negative log likelihood for the binary outcomes and mean squared error for the continuous outcome). We assessed prediction performance using five-fold CV-AUC for the binary outcomes and five-fold CV-R^2^ for the continuous outcome. We estimated intrinsic variable importance for AA features and feature groups using the difference in population AUCs for the bnAb regimens VRC07-523-LS + PGT121 and VRC07-523-LS + PGT121 + PGDM1400.

The code used to run SLAPNAP for each bnAb regimen is available on GitHub.^56^ The estimated prediction performance of the resulting PAR scores is provided in Table 1 and in Tables S2 and S3.

#### S4 Simulation: Improved comparisons of bnAB regimens by SLAPNAP

##### Continuous outcome

The simplest case for examining variance reductions is in the case of a continuous outcome (quantitative IC_80_). The results from SLAPNAP runs predicting IC_80_ for each bnAb or bnAb regimen in Table 1 are presented in the second column of Table S5. We define combination IC_80_ for *J* bnAbs according to combination IC_80_ = ${\left(\sum_{j=1}^{J}{IC}_{80,j}^{-1}\right)}^{-1}$.

For this simulation, for each bnAb in Table S5, we denote by *W* the log_10_ PAR score obtained from SLAPNAP and by *Y* the log_10_ (combination) IC_80_ readout. The untransformed PAR score is an estimator of IC_80_ in this case. We suppose that (*W*, *Y*) ∼ *N*(*μ*, *Σ*), where

$$\mu =\left(\mu_{1},\mu_{2}\right)\;and\;\Sigma =\left[\begin{array}{cc} \Sigma_{11} & \Sigma_{12}\\ \Sigma_{21} & \Sigma_{22} \end{array}\right]$$

are the mean vector and covariance matrix, respectively. For simplicity, we set μ_1_ = μ_2_ = μ^∗^, with μ^∗^ the mean log_10_ IC_80_ value in the CATNAP data^20^ for the specified bnAb (or bnAb regimen), and set $\Sigma_{11}$ = $\Sigma_{22}$ = $\Sigma^{*}$, with $\Sigma^{*}$ the variance of log_10_ IC_80_ in the CATNAP data for the specified bnAb (or bnAb regimen). Under this model, we can write

$$R^{2}=1-\frac{var\left(W\right)}{var\left(Y\right)}$$

$$=1-\frac{\sum_{12}^{2}\sum_{22}^{-1}}{\sum_{11}}$$

We can then generate data by specifying $\sum_{12}^{2}=\left(1-R^{2}\right)\sum_{22}\sum_{11}\text{,}$ using the point estimates of CV-*R*^2^ provided in Table S5 for the *R*^2^ value (and under the simplifying assumption made above, $\Sigma_{11}=\Sigma_{22}=\Sigma^{*}$.

For each bnAb or bnAb regimen under consideration, we generated 1000 random datasets according to the following specification:

1. We generated $n_{1}=1000$ iid copies $\left(W_{i},N_{i}\right)∼\;N\;\left(\mu^{*},\sum^{*}\right)$ , where again *Y* denotes the log_10_ (combination) IC_80_ values and *W* denotes the corresponding log_10_ PAR scores. We truncate the upper limit of *Y* at 1 (equivalent to an IC_80_ of 10), and do not truncate the upper limit of the PAR scores. We have found that IC_80_ > 10 μg/ml indicates that a virus is resistant to the bnAb (regimen). Thus, this cutoff provides the key information for discriminating viruses above a cutoff of 10, and our interest was in discriminating viruses below this cutoff.
2. We then generated ϵ×100% additional log_10_ PAR scores from this same distribution and suppress the IC_80_ measurement, where ϵ∈{0.5,1,2}.

We used the variable *R* to indicate whether *Y* was observed or not; *R*_*i*_ = 1 implies that *Y*_*i*_ was observed. The final dataset is then (*W*, *R*, *RY*). Note that in this example, λ=λ(w)=P(R=1|W=w)=P(R=1), since observing Y is independent of the PAR score. We generated 3000 datasets (*W*, *R*, *RY*) according to this specification, setting ϵ=2. From these datasets, we took 2000 datasets and randomly deleted observations to create the 1000 datasets for analysis of ϵ∈{0.5,1}.

For each randomly sampled dataset, our goal was to estimate the mean value of Y,θ≡E(Y); we compared estimating θ among those pseudoviruses with *R*_*i*_ = 1 (using *n*_*1*_ observations) to estimating θ using all pseudoviruses with a log_10_ PAR score (using *n =* (1 + ϵ) *n*_*1*_ observations). We first considered an estimator of θ that ignores those observations with *R*_*i*_ = 0:

(Equation 1) $$\theta_{n,1}:\frac{1}{n_{1}}\sum_{i=1}^{n}R_{i}Y_{i}\text{.}$$

We incorporated auxiliary information in estimating the mean using the method of Rotnitzky and Robins,^33^ and used code developed by Gilbert et al.^60^ In particular, the semiparametric efficient estimator of θ based on a sample of size *n* (denoted by $\theta_{n,2}$) solves the estimating equation $\sum_{i=1}^{n}U_{i}\left(\theta ,\lambda_{i}\right)=0$, where $\lambda_{i}=P\left(R_{i}=1|W_{i}\right)$ and

$$U_{i}\left(\theta ,\lambda_{i}\right)=\frac{R_{i}}{\lambda_{i}}\left(Y_{i}-\theta \right)-\frac{\left(R_{i}-\lambda_{i}\right)}{\lambda_{i}}\left\{E\left(Y|W_{i}\right)-\theta \right\}\text{.}$$

We estimated $E\left(Y|W_{i}\right)$ using ordinary least squares, denoting our estimator as $g_{n}:w\;\mapsto \;g_{n}\left(w\right)$, and then used the estimating equation above to obtain an estimator of *θ*. In our case, a consistent estimator of $\lambda_{i}$ is $\lambda_{n,i}=\frac{n_{1}}{n}$ for all *i*. In this special case, a closed form solution for $\theta_{n,2}$ exists:

(Equation 2) $$\theta_{n,2}=\frac{1}{n_{1}}\sum_{i=1}^{n}R_{i}\left(Y_{i}-g_{n}\left(W_{i}\right)\right)+\frac{1}{n}\;\sum_{i=1}^{n}g_{n}\left(W_{i}\right)$$

As mentioned above, this estimator has been shown to be both an unbiased and efficient estimator of the population mean.

We then computed the Monte-Carlo variance of the estimated means $\theta_{n,1}$ and $\theta_{n,2}$ over the 1000 replications, and estimated the relative efficiency of using the additional pseudoviruses by taking the ratio of the Monte-Carlo variance ignoring the additional pseudoviruses (the Monte-Carlo variance of $\theta_{n,1}$) to the Monte-Carlo variance using the additional pseudoviruses (the Monte-Carlo variance of $\theta_{n,2}$).

We performed a sensitivity analysis by keeping $\mu_{1}$ and $\Sigma_{11}$ as defined above but setting $\mu_{2}$ and $\Sigma_{22}$ equal to the observed values from the SLAPNAP predictions on CATNAP for the given bnAb regimen.

#### Binary outcome

We used a similar approach for binary outcomes to the approach outlined in the previous section. We now denote by *Y* the indicator that (combination) IC_80_ < 1 μg/mL, and denote by W the logit PAR score. In this setting, where we are predicting a binary outcome, the (untransformed) PAR score for a given pseudovirus is the predicted probability that the pseudovirus has (combination) IC_80_ < 1 μg/ml based on the AA sequence, and thus lies in [0, 1]; the logit PAR score then lies in (−∞,∞). We suppose that Y∼Bern(p), where *p* is the sample proportion with (combination) IC_80_ < 1 μg/ml for each bnAb in the CATNAP data, and that $W\;|\;Y=y\;∼\;N\left(\mu_{y},\sigma_{y}^{2}\right)$. Based on this specification, we can write (for two iid samples (*W*_1_, *Y*_1_) and (*W*_2_, *Y*_2_))

$$AUC=P\left(W_{1}<\;W_{2}|\;Y_{1}=0,Y_{2}=1\right)$$

$$=P\;\left(W_{2}-W_{1}>0|Y_{1}=0,Y_{2}=1\right)$$

Setting $Z=W_{2}-W_{1}$, we see that conditional on $\left(Y_{1},Y_{2}\right)$, *Z* has a normal distribution with mean *μ*_1_ −*μ*_0_ and variance $\sigma_{0}^{2}+\sigma_{1}^{2}$. Thus,

$$AUC=P\left(\frac{Z-\left(\mu_{1}-\mu_{0}\right)}{\sqrt{\sigma_{0}^{2}+\sigma_{1}^{2}}}>\;\frac{-\left(\mu_{1}-\mu_{0}\right)}{\sqrt{\sigma_{0}^{2}+\sigma_{1}^{2}}}\right)$$

$$\Rightarrow \;\frac{\left(\mu_{1}-\mu_{0}\right)}{\sqrt{\sigma_{1}^{2}+\sigma_{0}^{2}}}=\left(-1\right)\Phi^{-1}\left(1-AUC\right)$$

where Φ denotes the standard normal cdf. Thus, setting $\sigma_{1}^{2}=\sigma_{0}^{2}$ = 0.005 and *μ*_0_ = −0.32 (corresponding to a mean PAR score of 0.42 among those with *Y* = 0), we generated *n*_*1*_ iid copies of *W* and *Y* with *R* = 1, and generate ϵ × 100% iid copies of *W* with *R* = 0 (the additional logit PAR scores). The point estimates of CV-AUC are provided in Table S6. Again, the full data are (1 + ϵ)*n*_*1*_ iid copies of $\left(W_{i},R_{i},R_{i}Y_{i}\right)$.

For each randomly sampled dataset, our goal was next to estimate $\theta =E\left(Y\right)=P\left({IC}_{80}<1\;\mu g/ml\right)$; we again compared estimating *θ* among those pseudoviruses with *R*_i_ = 1 (using *n*_*1*_ observations) to estimating *θ* using all pseudoviruses with a logit PAR score (using *n =* (1 + ϵ)*n*_*1*_ observations). We used an identical estimation procedure to that described in the previous section, with one exception: we used a logistic regression model to estimate $E\left(Y|W_{i}\right)$. We then computed the Monte-Carlo variance of the estimated means over the 1000 replications as before, and estimated the relative efficiency of using the additional pseudoviruses by taking the ratio of the Monte-Carlo variance ignoring the additional pseudoviruses to the Monte-Carlo variance using the additional pseudoviruses.

We also computed the bias of both estimators – from Equation 1 (augmented = FALSE) and S2 (augmented = TRUE) for estimating the true mean θ for both outcome types. The results are displayed in Figure S3.

#### S5 Data analysis: Improved comparisons of bnAB regimens by SLAPNAP

In this analysis, we examined the variance reduction achieved by augmenting sequences with information in CATNAP with additional Env sequences from the LANL database.

For each bnAb regimen and outcome considered in Tables S2 and S3, we analyzed the data as follows:

1. Loaded the SLAPNAP predictor from Section S3 corresponding to the given bnAb regimen and outcome; denoted the predictor by $f_{n}=l\;\mapsto \;f_{n}\left(l\right)$, where l encodes Env sequence information.
2. Estimated E(Y|W) using the data input to SLAPNAP and the SLAPNAP predictions $w=f_{n}\left(l\right)$ based on the CATNAP data; denoted the predictor $g_{n}:w\;\mapsto \;g_{n}\left(w\right)$, as in Section S4(for continuous outcomes, linear regression was used; for binary outcomes, logistic regression was used).

3. For each country *c* with at least 30 pseudoviruses in CATNAP:
  (a) $X_{c}$ denoted the combined CATNAP and LANL data from this country, $Y_{c}$ denoted the observed log_10_ (combination) IC_80_ or indicator that (combination) IC_80_ < 1 μg/ml, and $R_{c}$ denoted the binary vector of whether each sequence was observed in CATNAP;
  (b) Obtained predictions $W_{c}$ based on the SLAPNAP predictor $f_{n}$ and the Env sequence data $X_{c}$. Set $D_{c}=\left(W_{c},R_{c},R_{c}Y_{c}\right)$;
  (c) Obtained predictions $g_{n}\left(W_{c}\right)$ based on the regression estimator $g_{n}$ and the country-specific predictions;
  (d) Estimated $\lambda_{c}=\frac{\#\left\{sequences\;in\;CATNAP\right\}}{\#\left\{sequences\;in\;LANL\right\}}$;
  (e) Obtained estimate $\theta_{n,1}$ of *θ* (the mean outcome value, as in Section S4) using only data in CATNAP (Equation 1);
  (f) Obtained estimate $\theta_{n,2}$ of *θ* using data in CATNAP and LANL, using Equation 2;
  (g) Obtained a non-augmented 95^th^ percentile bootstrap confidence interval for *θ*: drew 5000 bootstrap datasets ${\left\{D_{c,b}\right\}}_{b=1}^{5000}$. On each bootstrap dataset, $g_{n}$ was used to predict using $W_{c,b}\text{;}$ an estimate of $\theta_{n,1,b}$ was then obtained using data with $R_{c,b}=1$, taking the 2.5^th^ and 97.5^th^ percentiles of $\theta_{n,1,b}$;
  (h) Obtained an augmented 95^th^ percentile bootstrap confidence interval for *θ*: drew 5000 bootstrap datasets ${\left\{D_{c,b}\right\}}_{b=1}^{5000}$. On each bootstrap dataset, $g_{n}$ was used to predict using $W_{c,b}\text{;}$ an estimate of $\theta_{n,2,b}$ was then obtained the entire dataset, taking the 2.5^th^ and 97.5^th^ percentiles of $\theta_{n,2,b}$;
  (i) Computed the width of each 95% CI, obtaining $w_{1}$ and $w_{2}$, corresponding to the width of the 95% CI from (g) and the 95% CI from (h), respectively;
  (j) Computed the relative efficiency (RE) as the ratio of the squared non-augmented CI width to the squared augmented CI width, i.e., RE = $w_{1}^{2}/w_{2}^{2}$;
  (k) Computed the relative efficiency bound (i.e., information we can gain using observations in both LANL and CATNAP),
    $$REB=\frac{\#\left\{sequences\;available\;in\;both\;LANL\;and\;CATNAP\right\}}{\#\left\{sequences\;in\;CATNAP\right\}}\text{;}$$
  (l) Computed the bounded relative efficiency (BRE), defined as the minimum of the results of steps (j) and (k), i.e., BRE = min(RE, REB).

We display the results in a two-row plot, with the rows corresponding to continuous (combination) IC_80_ and the binary indicator that (combination) IC_80_ < 1 μg/ml. The horizontal axis of each plot is the proportion of additional information in LANL compared to CATNAP, defined as

$$\frac{\#\left\{sequences\;in\;LANL\right\}-\#\left\{sequences\;in\;CATNAP\right\}}{\#\left\{sequences\;in\;LANL\right\}}\text{,}$$

and the vertical axis is the bounded relative efficiency. We choose to display bounded relative efficiency because it is mathematically possible to observe large relative efficiencies, but the relative efficiency bound (defined using the number of sequences in both LANL and CATNAP, which allows us to link the two datasets) provides a meaningful limit on the relative efficiency we should expect to observe in practice.

#### S6 Simulation: SLAPNAP improves sieve analysis in bnAB efficacy trials

The goal of this simulation was to see if SLAPNAP improved sieve analysis; in particular, we wanted to see if using SLAPNAP resulted in improved power for detecting sieve effects. For simplicity, we used VRC01 to define residues that confer susceptibility or resistance for this simulation, and considered an Antibody Mediated Prevention (AMP) trial-like design with only the high-dose arm.^5^ In this simulation, we assumed that we were using data from a trial assessing the prevention efficacy of a combination bnAb regimen *R* (testing a CD4 binding site-targeting bnAb) that was expected to have higher prevention efficacy than that observed in AMP.

Suppose that we randomly assigned study participants in a 1:1 allocation to placebo or the combination bnAb regimen *R*, letting *A* ∈ {0, 1} denote the treatment assignment (0 = placebo, 1 = assignment to *R*). Suppose further that each participant in the trial had an underlying time to HIV-1 infection diagnosis (denoted by *T*), and a corresponding genotype for the acquired HIV-1 virus. We let $S=\left(S_{1},\ldots ,S_{J}\right)$ denote a binary indicator vector, where $S_{j}$ is the indicator that the Env sequence has the putative susceptibility-conferring residue present at amino acid (AA) position *j* in the Env protein, and *J* is the number of HXB2 positions in the Env protein that exhibit sufficient residue variability. We defined sufficient residue variability at a given position by at least 4 primary endpoint cases from AMP having a sequence with a minority residue at that position, pooled over both AMP trials (harmonized with the AMP sieve analysis). Based on AMP, there were 414 AA sites that exhibited sufficient variability (i.e., *J* = 414). More specifically, we assumed that

A∼Bern(0.5)

$$S_{j}\;∼\;Bern\left(\gamma_{0,j}\right)\;for\;j\in \left\{1,\ldots ,J\right\}\text{.}$$

We assumed here that $S_{1},\ldots ,S_{J}$ are mutually independent; while this is not true in general due to covariability of AA positions, this fact does not impact the simulation design, as we show below. In Table S7, we list positions in the Env gp120 region identified as important for the AMP sieve analysis with enough variability in the AMP data, along with the corresponding susceptible genotype (residue). We defined $\gamma_{0,j}$ to be the proportion of AMP placebo-arm participants with the putative susceptibility-conferring residue at AA position *j*. Table S7 also contains the values of $\gamma_{0,j}$ for the pre-identified positions. For positions in gp120 outside of this list of pre-identified positions, we selected a ‘putative susceptibility-conferring residue’ by identifying the residue observed to be most frequent among VRC01-susceptible viruses vs. VRC01-resistant viruses in CATNAP, and then defined the corresponding $\gamma_{0,j}$ as the proportion of this residue among AMP placebo-arm participants.

We assumed that *R* had an overall prevention efficacy of 0.7. We further assumed that for the positions given in Table S7, there may be greater prevention efficacy of *R* against viruses with a putative susceptibility-conferring residue at a given position than against viruses with another residue at the given position. This latter assumption can be formalized using the language of sieve analysis. We defined a sieve effect at AA position *j* as differential prevention efficacy (PE) at that position; more specifically, we conducted a hypothesis test of

$$H_{0}:PE\left(S_{j}=1\right)=PE\left(S_{j}=0\right)\;versus\;H_{1}:PE\left(S_{j}=1\right)\neq PE\left(S_{j=0}\right)\text{.}$$

For each position *j*, we defined the overall PE as a function of the PE of a susceptible and other genotype at position *j*, i.e.,

(Equation 3) $$\log\left\{1-PE\left(overall\right)\right\}=\gamma_{0,j}\;\log\left\{1-PE\left(S_{j}=1\right)\right\}+\left(1-\gamma_{0,j}\right)\log\left\{1-PE\left(S_{j}=0\right)\right\}$$

To simplify the simulation, we assumed the absence of sieve effects at all AA positions except for AA position 230. This position was observed to have a sieve effect with respect to VRC01 in one of the AMP trials (HVTN 704/HPTN 085), where the presence of residue D at position 230 was found to confer resistance to VRC01 (estimated 64% PE based on not residue D at 230 vs. estimated -24% PE based on residue D at 230). We initially assumed that $PE\left(S_{230}=0\right)=0$. Following Equation 3, under this assumption $PE\left(S_{230}=1\right)\approx 0.89$.

Next, we let $T_{0}$ and $T_{1}$ denote the latent cause-specific time to HIV-1 infection diagnosis with a virus with $S_{230}=0$ and $S_{230}=1$, respectively, and let *T* denote the latent HIV-1 infection diagnosis time with $T=\min\left\{T_{0},T_{1}\right\}$. More specifically, we modeled the latent cause-specific HIV-1 infection diagnosis times as

$$T_{0}\;|\;A=a∼\mathrm{Exp}\left(\lambda \;\exp\left[\log\left(1-\gamma_{0,230}\right)+a\;\log\left\{1-PE\left(S_{230}=0\right)\right\}\right]\right)$$

$$T_{1}\;|\;A=a∼\mathrm{Exp}\left(\lambda \;\exp\left[\log\;\gamma_{0,230}+a\;\log\left\{1-PE\left(S_{230}=1\right)\right\}\right]\right)$$

$$T=\min\left\{T_{0},T_{1}\right\}$$

Under the above model for the latent HIV-1 infection diagnosis times we can write the following hazards (with $h_{j}\left(a\right)$ denoting the cause-specific hazard of HIV-1 infection with genotype j for $S_{230}=j$ in arm A = a):

$$h_{0}\left(A=0\right)=\lambda \left(1-\gamma_{0,230}\right)$$

$$h_{1}\left(A=0\right)=\lambda \gamma_{0,230}$$

$$h_{0}\left(A=1\right)=\lambda \left(1-\gamma_{0,230}\right)\left\{1-PE\left(S_{230}=0\right)\right\}$$

$$h_{1}\left(A=1\right)=\lambda \gamma_{0,230}\left\{1-PE\left(S_{230}=1\right)\right\}\text{.}$$

Under the assumption that $PE\left(S_{230}=0\right),h_{0}\left(A=1\right)=\lambda \left(1-\gamma_{0,230}\right)=h_{0}\left(A=0\right)$, which aligns precisely with our assumptions above that prevention efficacy based on the resistant genotype at 230 is zero. We assumed three different baseline hazards among those with $S_{230}=0:\lambda \in \left\{.03,.018,.006\right\}$, corresponding to three different incidence rates. In each of the three settings, the baseline hazard was calibrated so that we expected to see approximately 88 HIV-1 infection diagnosis endpoints combined over the two arms over the 24-month follow-up period, which is the number of events needed to have 90% power to detect 50% overall prevention efficacy versus a null hypothesis of 0% overall prevention efficacy in a trial designed with a 1:1 allocation of study participants to the placebo or bnAb arms. Gilbert61 considered this design as a potential sequel phase 2b design to the AMP trials. These baseline hazards imply sample sizes n∈{2071,4141,12422} (see Table 2 in Gilbert^61^).

We then defined the following censoring and observation processes:

C∼Unif(0,c)

Δ=I(T≤C&T≤24months)

Y=ΔT+(1−Δ)min{C,24months}

$$V=\Delta S_{230}\text{,}$$

Where *C* denotes the censoring time, which is independent of *T*; Δ denotes the indicator that we have observed an HIV-1 infection diagnosis event before the end of the study (at 24 months for primary outcome adjudication) or censoring; *Y* denotes the observation time; and *V* denotes the observed “mark”, i.e. the presence of a susceptibility-conferring residue at site 230. Note that in participants for whom we observe Δ = 0, i.e., the participant does not acquire HIV-1 infection, *Y* is the minimum of the censoring time and end of the study and *V* is not defined. We assumed a yearly censoring rate of 10%, implying that c = (365 x 2)/0.1.

We used Cox modeling [Lunn and McNeil (1995)]43, implemented in the R package sievePH) to estimate differential prevention efficacy. For each of 1000 Monte-Carlo replications, we generated data according to the following specification. For a given baseline hazard λ and corresponding sample size *n*, we first generated *n* independent observations of

A∼Bern(0.5)

$$T_{0}∼\mathrm{Exp}\left(\lambda \;\exp\left[\log\left(1-\gamma_{0,230}\right)+a\;\log\left\{1-PE\left(S_{230}=0\right)\right\}\right]\right)$$

$$T_{1}∼\mathrm{Exp}\left(\lambda \;\exp\left[\log\;\gamma_{0,230}+a\;\log\left\{1-PE\left(S_{230}=1\right)\right\}\right]\right)$$

$$T=\min\left\{T_{0},T_{1}\right\}$$

$$S_{230}=I\left\{T=T_{1}\right\}$$

$$S_{j}\;∼\;Bern\left(\gamma_{0,j}\right)\;for\;j\in \left\{1,\ldots ,J\right\}\;\setminus \;\left\{230\right\}\;mutually\;independent$$

C∼Unif(0,c)

We then set $\Delta =I\left(T\leq C\;\&\;T\leq 24\;months\right),Y=\Delta T+\left(1-\Delta \right)\min\left\{C,24\;months\right\},and\;V=\Delta S_{230}$, where again if Δ=0 then *V* is missing.

For a given dataset, we considered two different sieve analysis strategies. The first is to apply a standard Lunn and McNeil test for differential PE at each Env gp120 site with sufficient variability *j* = 1, …, *J* with two-sided level 0.05; we defined a detection as the resulting p-value from this test being less than the family-wise error rate using a multiplicity-adjusted threshold of 0.05, implemented using a Holm-Bonferroni adjustment. We call this a “site-scanning” sieve analysis. The second is a “priority” sieve analysis, where we apply a standard Lunn and McNeil test for differential PE at each of the important high-variability sites listed in Table S7, and define a detection as the resulting p-value from this test being less than the family-wise error rate multiplicity-adjusted threshold of 0.05, again using a Holm-Bonferroni adjustment.

The priority sieve analysis has the opportunity to provide greater power because we adjust for a smaller number of hypothesis tests, if indeed the SLAPNAP procedure for identifying sites most likely to impact *in vivo* vaccine efficacy has some predictive power. We computed the power as the Monte-Carlo average number of true detections across the 1000 replications and repeated the process of generating 1000 Monte-Carlo replications for each assumed baseline hazard and corresponding sample size. Comparing the power of the site-scanning approach to the priority approach provides a useful description of how much SLAPNAP aids in sieve analysis.

To better understand power as a function of the effect size, we also generated data under several different hypothesized values of $PE\left(S_{230}=0\right)\text{.}$ The corresponding parameter values used in the simulations are provided in Table S8. The case $PE\left(S_{230}=0\right)=PE\left(S_{230}=1\right)=0.7$ corresponds to the sieve null hypothesis, in which case the power of the sieve analyses should be controlled at the type I error rate (0.05). The case $PE\left(S_{230}=0\right)=0$ should result in the highest power for detecting sieve effects.

### Additional resources

NCT02165267, NCT02568215, NCT02716675, NCT02797171, NCT03387150, NCT03735849, NCT04212091, NCT05184452, NCT04212091, NCT03928821, NCT03928821, NCT05184452 (trials listed in Table S1).

## Data Availability

• This paper analyzes existing, publicly available data. The data are available at https://hiv.lanl.gov/catnap.^20^ The data used in the analyses are compiled using code deposited at Zenodo.^50^ The DOI is listed in the key resources table. • All original code has been deposited at Zenodo and is publicly available. The DOI is listed in the key resources table. • Any additional information required to reanalyze the data reported in this paper is available from the lead author upon request.

## Supplemental information

Document S1. Figures S1–S9 and Tables S1–S3 and S5–S8

Table S4. Complete information on cross-validated prediction performance for nine different bnAbs or combination bnAb regimens, related to Table 1 For each bnAb (VRC01, VRC07-523-LS, PGT121, VRC26.25) or combination bnAb regimen (VRC07-523-LS + PGT121, VRC07-523-LS + PGDM1400, VRC07-523-LS + 10–1074, VRC07-523-LS + PGT121 + PGDM1400, VRC01/ PGDM1400/10e8v4), the table provides eight different metrics for assessing cross-validated prediction performance (sensitivity, specificity, positive predictive value [PPV], negative predictive value [NPV], F1 score [harmonic mean of sensitivity and PPV], F0.5 score [weighted harmonic mean of sensitivity and PPV, with more weight given to PPV], accuracy, and Matthews correlation coefficient [MCC]) at each quantile of predicted risk, along with the AUC and Brier score. Susceptibility is defined as (combination) IC_80_ < 1 μg/mL, where for *J* bnAbs, combination IC_80_ = ${\left(\sum_{j=1}^{J}{IC}_{80,j}^{-1}\right)}^{-1}$ and multiple susceptibility is defined as IC_80_,_*j*_ < 1 for at least 1 bnAb (where applicable).
