# Supplementary material for: Application of the SLAPNAP statistical learning tool to broadly neutralizing antibody HIV prevention research
Source: iScience. 2023 Aug 9;26(9):107595. doi: 10.1016/j.isci.2023.107595 (PMC10466901; doi:10.1016/j.isci.2023.107595)
Supplement: Document S1. Figures S1–S9 and Tables S1–S3 and S5–S8 [file mmc1.pdf]

iScience, Volume *26*

Supplemental information

Application of the SLAPNAP statistical learning tool to broadly neutralizing antibody HIV prevention research

Brian D. Williamson, Craig A. Magaret, Shelly Karuna, Lindsay N. Carpp, Huub C. Gelderblom, Yunda Huang, David Benkeser, and Peter B. Gilbert

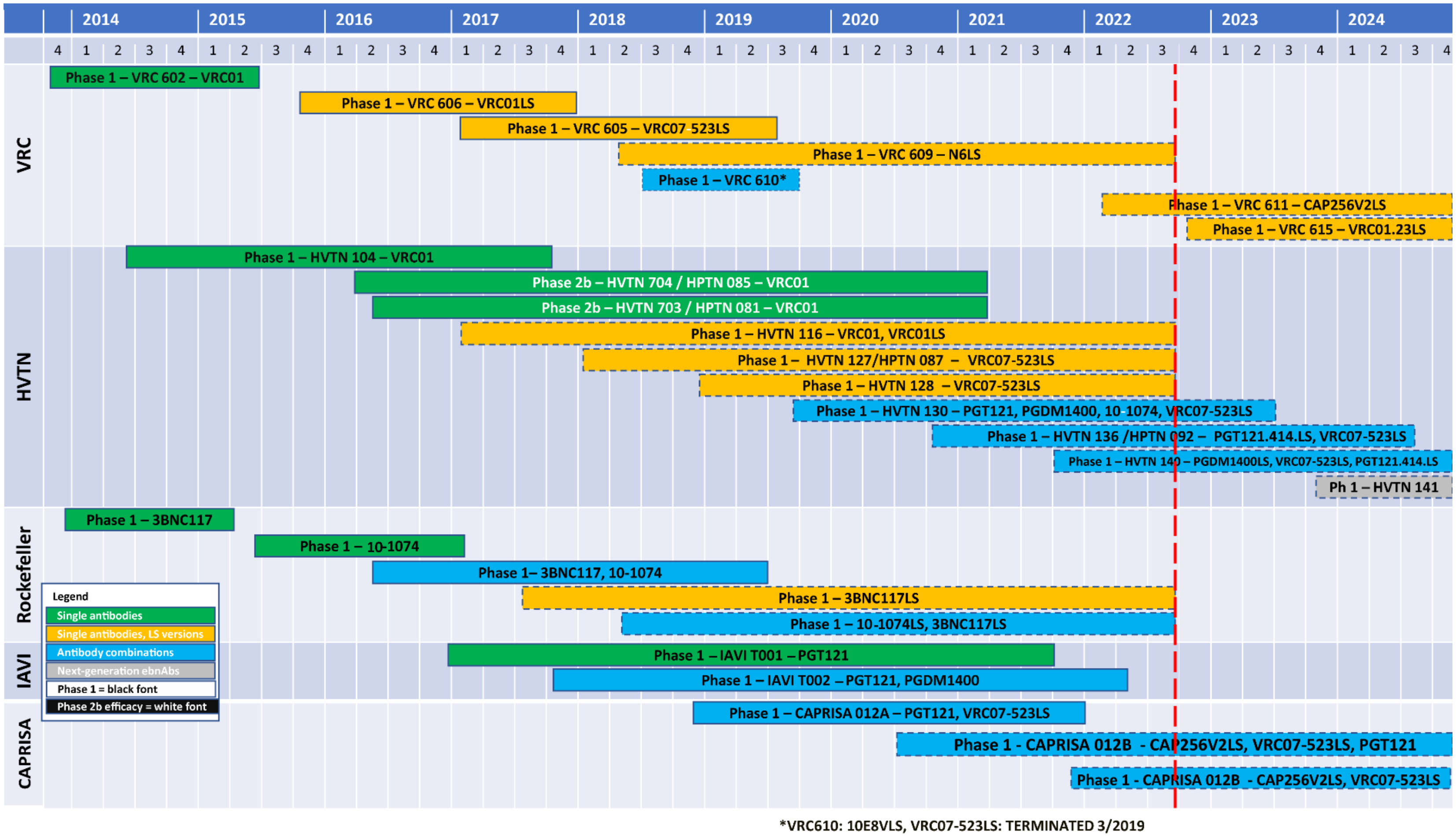

Figure S1. Overview of HIV broadly neutralizing monoclonal antibody (bnAb) clinical trials in persons living without HIV for the time frame 2013-2024, Related to Table 1. The horizontal bars extend from the date of trial start through to the date of the primary publication. Bars with solid outlines represent trials whose primary results have been published. Bars with dashed outlines represent ongoing or yet-to-have started trials. All data past the vertical dashed red line are projected. For space reasons, the following HIV Prevention Trials Network (HPTN) names could not be included in the schematic: HIV Vaccine Trials Network (HVTN) 130/HPTN 089, HVTN 140/HPTN 101, HVTN 141/HPTN 105. CAPRISA = Centre for the Aids Programme of Research in South Africa, IAVI = International AIDS Vaccine Initiative, VRC = Vaccine Research Center.

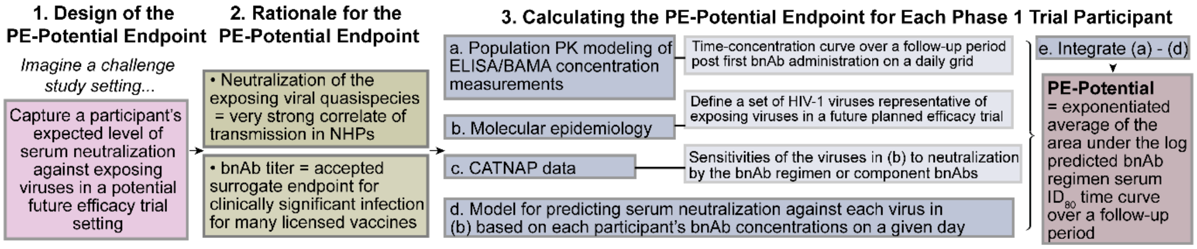

Figure S2. Ranking of combination bnAb regimens by the prevention efficacy potential (PE-Potential) endpoint, Related to Table 1. ID_{80} in the figure is inhibitory dilution of a bnAb recipient's serum sample that yields 80% reduction of HIV-1 infection of target cells in a neutralization assay. ID_{80} for a bnAb regimen recipient at a given day and a given potential exposing virus on that day is predicted by combination PT80 defined in Gilbert et al. [S1], calculated by combining across each individual bnAb PT80 in the bnAb regimen, where individual bnAb PT80 is the concentration of the bnAb on the day divided by the IC_{80} of the bnAb against the virus.

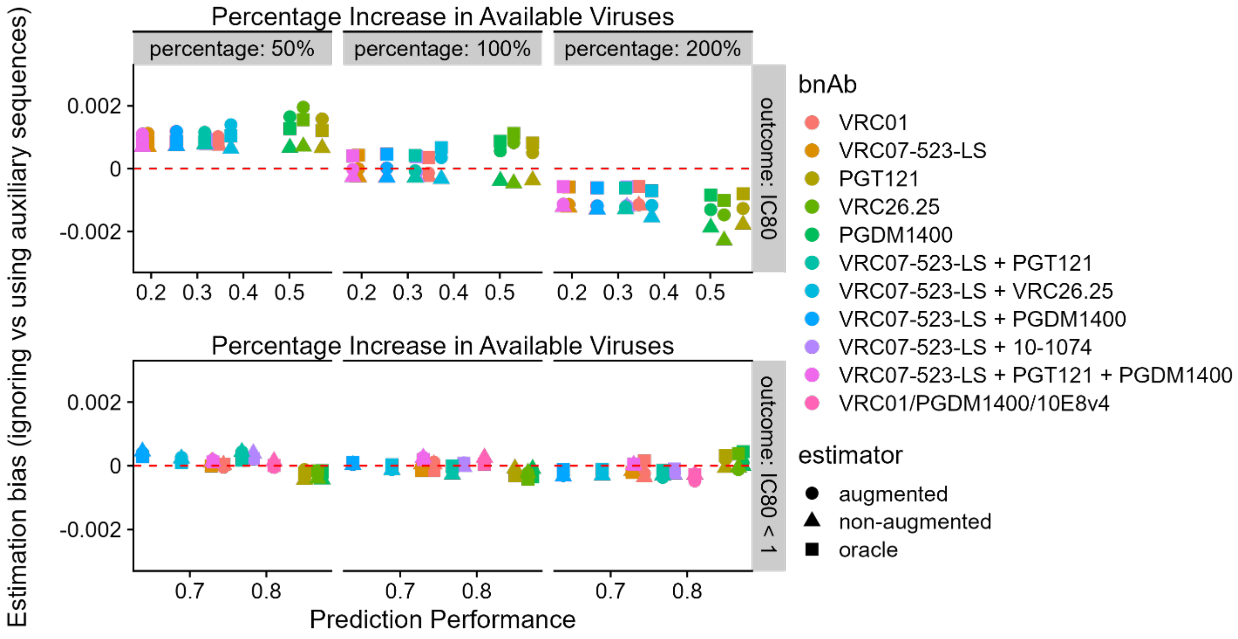

Figure S3. Estimation bias (mean difference between the estimated outcome and the true outcome, taken over 1000 simulated replicates) versus estimated SLAPNAP-prediction performance for each bnAb regimen listed in Table S1, Related to Figure 1. Top row: Prediction of IC_{80}. Bottom row: Prediction of binary IC_{80} < 1 μg/ml. Columns denote the percentage increase in the number of viruses included in the SLAPNAP-augmented approach when adding viruses with data on Env sequence data only to viruses with data on both Env sequence and IC_{80}. The bnAb regimens are differentiated by color, while the estimator (non-augmented, augmented, or oracle) is differentiated by shape; each point represents the average bias over 1000 simulated replicates.

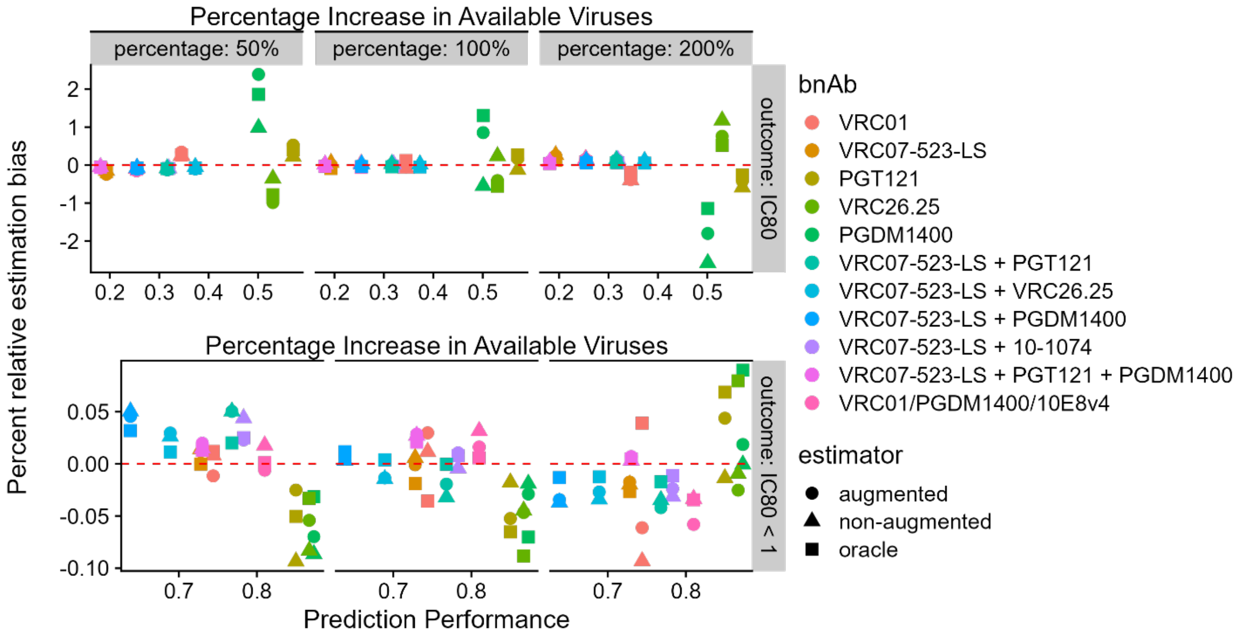

Figure S4. Percent relative estimation bias [mean over 1000 simulated replicates of {(estimated neutralization value – true neutralization value) / true neutralization value} x 100%] versus estimated SLAPNAP-prediction performance for each bnAb regimen listed in Table S1, Related to Figure 1. Top row: Prediction of IC_{80}. Bottom row: Prediction of binary $IC_{80} < 1$ μg/ml. Columns denote the percentage increase in the number of viruses included in the SLAPNAP-augmented approach when adding viruses with data on Env sequence data only to viruses with data on both Env sequence and IC_{80}. The bnAb regimens are differentiated by color, while the estimator (non-augmented, augmented, or oracle) is differentiated by shape; each point represents the average percent relative bias over 1000 simulated replicates.

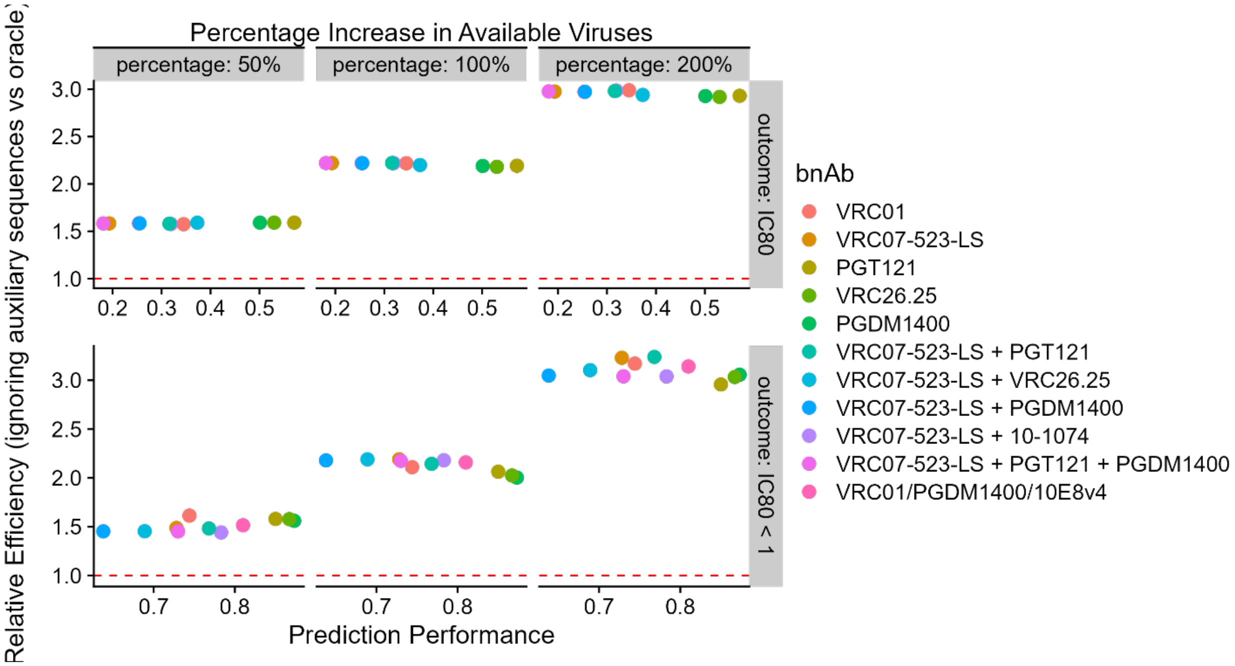

Figure S5. Relative efficiency (ratio of the sample variances of the estimated mean outcome value for the Reference approach vs. oracle approach, taken over 1000 simulated replicates) versus estimated SLAPNAP-prediction performance for each bnAb regimen listed in Table S1, Related to Figure 1. Top row: Prediction of IC80. Bottom row: Prediction of binary IC80 < 1 μg/ml. Columns denote the percentage increase in the number of viruses included in the oracle approach. The bnAb regimens are differentiated by color; each point represents the ratio of the Monte-Carlo variances taken over 1000 simulated replicates.

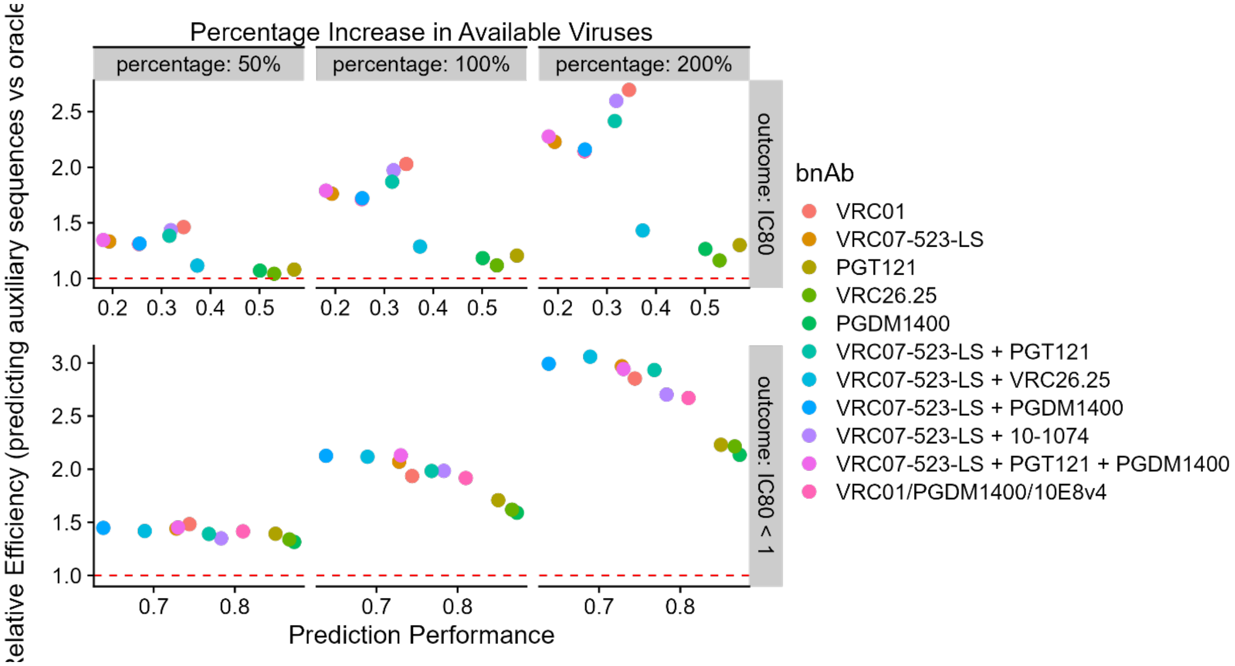

Figure S6. Relative efficiency (ratio of the sample variances of the estimated mean outcome value for the SLAPNAP-augmented approach vs. oracle approach, taken over 1000 simulated replicates) versus estimated SLAPNAP-prediction performance for each bnAb regimen listed in Table S1, Related to Figure 1. Top row: Prediction of IC80. Bottom row: Prediction of binary IC80 < 1 μg/ml. Columns denote the percentage increase in the number of viruses included in both approaches. The bnAb regimens are differentiated by color; each point represents the ratio of the Monte-Carlo variances taken over 1000 simulated replicates.

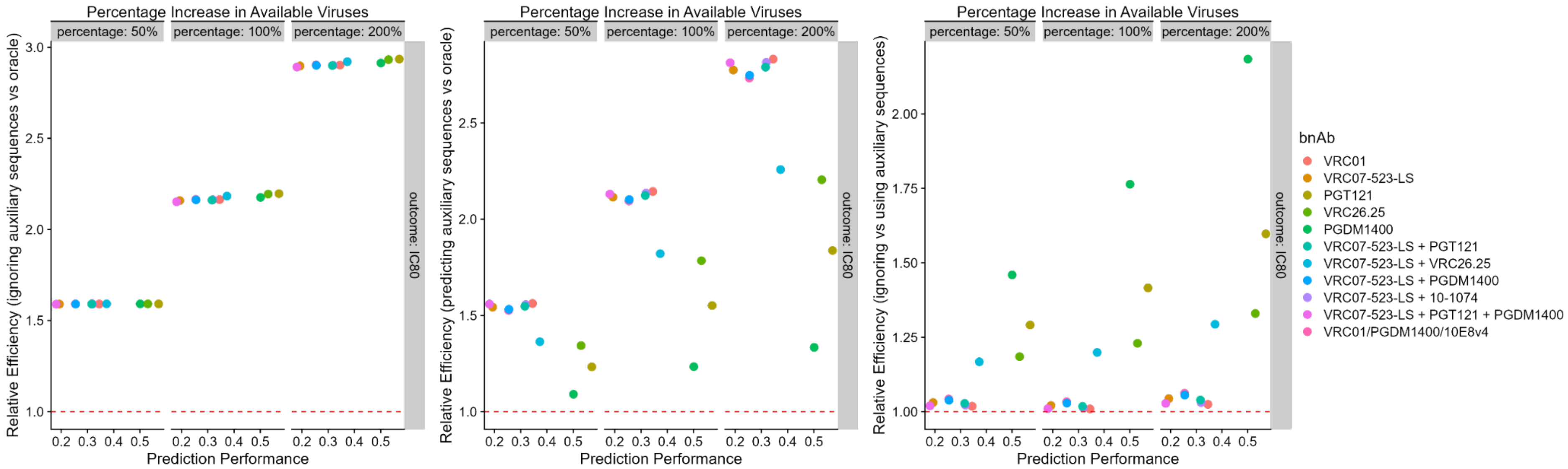

Figure S7. Relative efficiency (ratio of the sample variances of the estimated mean outcome value between two approaches, taken over 1000 simulated replicates) versus estimated SLAPNAP-prediction performance for each bnAb regimen listed in Table S1 for predicting IC80 from a sensitivity analysis where the observed mean and variance of IC80 and the SLAPNAP predictions were used to generate data rather than generating from a common mean and variance, Related to Figure 1. Left: reference approach vs oracle approach. Center: SLAPNAP-augmented approach vs oracle approach. Right: reference approach vs SLAPNAP-augmented approach. Columns denote the percentage increase in the number of viruses included in both approaches. The bnAb regimens are differentiated by color; each point represents the ratio of the Monte-Carlo variances taken over 1000 simulated replicates.

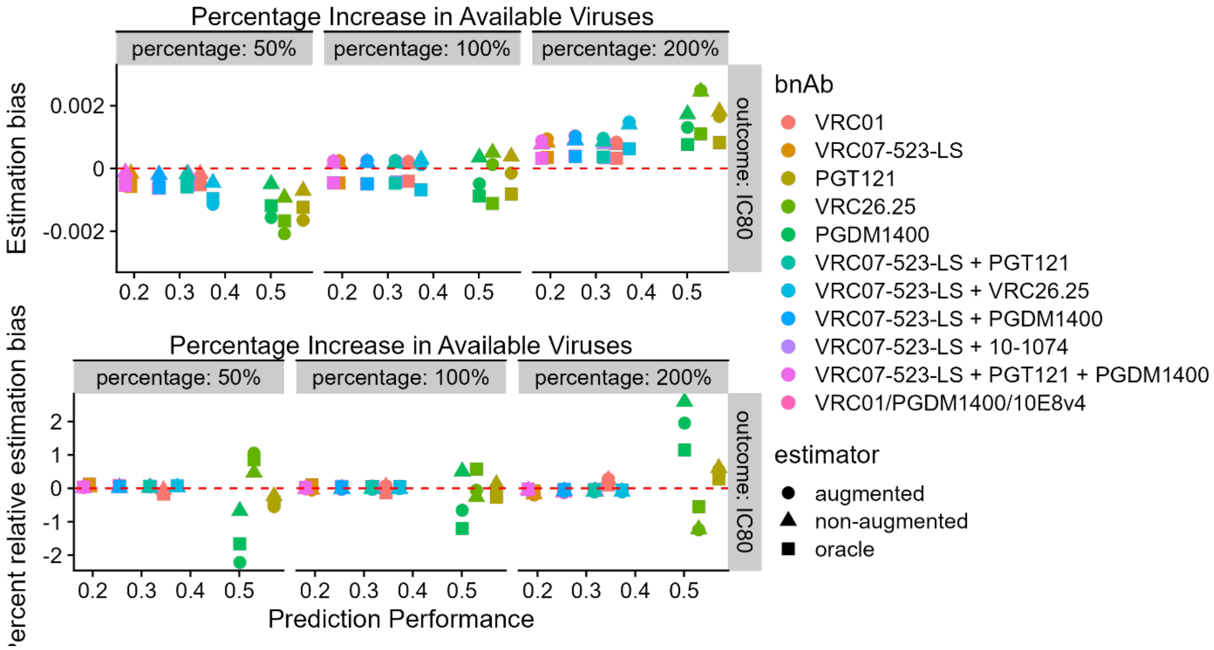

Figure S8. Estimation bias (mean difference between the estimated outcome and the true outcome, taken over 1000 simulated replicates) and percent relative estimation bias [mean over 1000 simulated replicates of {(estimated neutralization value – true neutralization value) / true neutralization value} x 100%] versus estimated SLAPNAP-prediction performance for each bnAb regimen listed in Table S1, from a sensitivity analysis where the observed mean and variance of IC80 and the SLAPNAP predictions were used to generate data rather than generating from a common mean and variance, Related to Figure 1. Top row: Estimation bias for prediction of IC_{80}. Bottom row: Percent relative estimation bias for prediction of IC_{80}. Columns denote the percentage increase in the number of viruses included in the SLAPNAP-augmented approach when adding viruses with data on Env sequence data only to viruses with data on both Env sequence and IC_{80}. The bnAb regimens are differentiated by color, while the estimator (non-augmented, augmented, or oracle) is differentiated by shape; each point represents the average over 1000 simulated replicates.

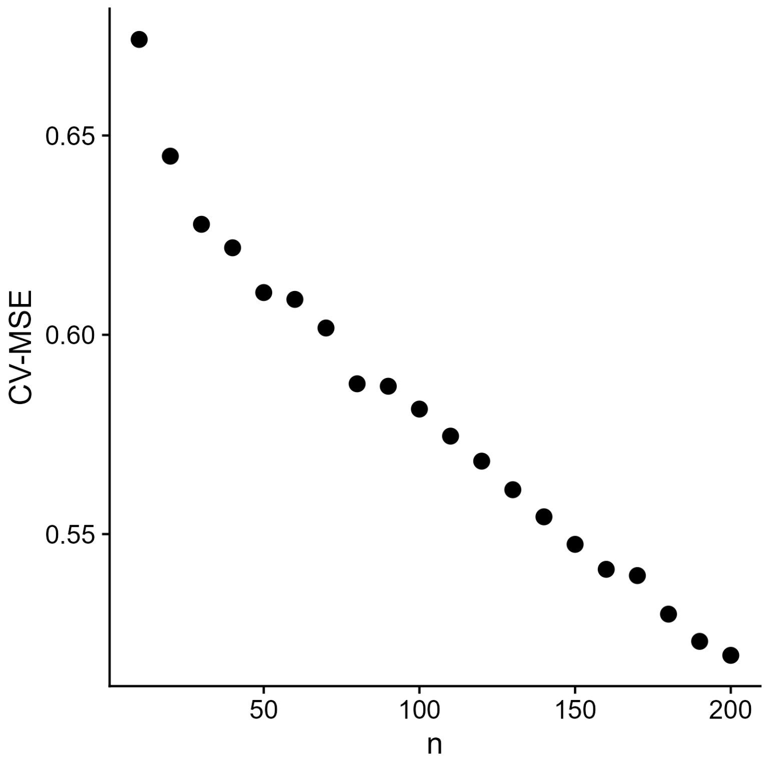

Figure S9. Cross-validated mean-squared error (CV-MSE) of a lasso regression model for predicting $\log_{10} IC_{80}$ based on a sample of size n from the CATNAP data for VRC01, Related to STAR Methods.

Table S1. bnAb regimens undergoing DAIDS HVTN and HVTN/HPTN clinical testing as of October 2022¶ and numbers of (pseudo)viruses with Env gp160 sequence data in the LANL HIV sequence data base that also have neutralization IC_{80} data in the CATNAP data base, Related to Table 1.

		Numbers of Env pseudoviruses with IC_{80} data in the CATNAP data base for neutralization by the given bnAb or by each of the constituent bnAbs in a multispecific bnAb or combination bnAb regimen		
bnAb regimen	DAIDS HVTN and HVTN/HPTN Clinical Trial(s)	All	All ≥ 2005	Clade C SSA‡ ≥ 2005
**1-bnAb regimen (s)**				
VRC01<br><br><br><br><br><br>VRC01, VRC01-LS*	HVTN 104 [phase 1, completed [S2] (NCT02165267)<br>HVTN 703/HPTN 081 [phase 2b, completed [S3] (NCT02568215)<br>HVTN 704/HPTN 085 [phase 2b, completed [S3] (NCT02716675)<br>HVTN 116 (phase 1, completed, NCT02797171)	572	343	158
VRC07-523-LS	HVTN 127/HPTN 087 (phase 1, completed, NCT03387150)<br>HVTN 128 (phase 1, completed, NCT03735849)	400	210	157
PGT121.414.LS*	HVTN 136/HPTN 092 (phase 1, ongoing, NCT04212091)	542	336	158
CAP256V2LS*¶	HVTN 138/HPTN 038 (did not proceed)¶	400	210	157
PGDM1400LS*	HVTN 140/HPTN 101 (phase 1, ongoing, NCT05184452)	521	331	157
**2-bnAb regimens/bispecific bnAbs**				
PGT121.414.LS* + VRC07-523-LS†	HVTN 136/HPTN 092 (phase 1, ongoing, NCT04212091)	400	210	157
CAP256V2LS* + VRC07-523-LS	HVTN 138/HPTN 098 (phase 1, in development, NCT pending)	400	210	157
PGDM1400 + VRC07-523-LS<br>PGT121 + VRC07-523-LS†<br>10-1074 + VRC07-523-LS	HVTN 130/HPTN 089 (phase 1, completed, NCT03928821) (manuscript submitted)	400	210	157
**3-bnAb regimens/trispecific bnAbs**				
PGDM1400 +<br>PGT121 +<br>VRC07-523-LS	HVTN 130/HPTN 089 (phase 1, completed, NCT03928821)	400	210	157
PGDM1400LS* +<br>PGT121.414.LS* +<br>VRC07-523-LS	HVTN 140/HPTN 101 (phase 1, ongoing, NCT05184452)	400	210	157
VRC01/PGDM1400/10e8v4¶	HVTN 129/HPTN 088 (did not proceed)¶	205	48	13

* While VRC01-LS, PGT121.414.LS, PGDM1400LS, and CAP256V2LS do not have data in CATNAP, the non-LS parental versions (VRC01, PGT121, PGDM1400, and VRC26.25, respectively) do and their data are used here. CAP256VLS additionally contains the K127A mutation, which is not hypothesized to affect neutralization.
¶ Two bnAbs are included in this table that were planned for clinical testing in DAIDS HVTN and HVTN/HPTN clinical trial(s) at the time of drafting the manuscript and were hence included in the analyses, but which did not

ultimately advance and hence are not undergoing testing, nor are planned to be tested in future, in the Networks as of October 2022. These two bnAbs are: the parental bnAb of CAP256V2LS (VRC26.25) and the trispecific bnAb VRC01/PGDM1400/10e8v4.

†Since CATNAP does not differentiate PGT121 from PGT121.414.LS (yet), these two rows are identical in terms of data retrieved from CATNAP.

‡SSA is an abbreviation for Sub-Saharan Africa.

Note: HVTN 141/HPTN 105 (phase 1) is projected to initiate in Q4 2023 and is not included in this table.

Table S2. Prediction performance (sensitivity, specificity, positive predictive value [PPV], negative predictive value [NPV], F1 score [harmonic mean of sensitivity and PPV], F0.5 score [weighted harmonic mean of sensitivity and PPV, with more weight given to PPV], accuracy, Matthews correlation coefficient [MCC], area under the receiver operating characteristic curve [AUC], and Brier score) *at the quantile of predicted risk that maximized the F1 score* when predicting the binary outcomes for each broadly neutralizing antibody (bnAb) or bnAb regimen from Table 1, Related to Table 1. Susceptibility is defined as (combination) IC_{80} < 1 μg/ml, where for J bnAbs, combination IC_{80} = $\left(\sum_{j=1}^{J} \mathrm{IC}_{80,j}^{-1}\right)^{-1}$ and multiple susceptibility is defined as $IC_{80,j}$ < 1 for at least 1 bnAb (where applicable).

bnAb (or bnAb regimen)	Outcome	Quantile	Cutoff	Sensitivity	Specificity	PPV	NPV	F1	F0.5	Accuracy	MCC	AUC	Brier
VRC01	Susceptibility	0.43	0.3	0.78	0.56	0.54	0.81	0.64	0.57	0.65	0.35	0.74	0.19
PGT121	Susceptibility	0.43	0.38	0.88	0.7	0.72	0.87	0.79	0.74	0.78	0.58	0.84	0.36
VRC01/PGDM1400/ 10e8v4	Susceptibility	0.03	0.47	0.99	0.16	0.84	0.93	0.91	0.87	0.84	0.34	0.79	0.2
VRC07-523-LS	Susceptibility	0.03	0.57	0.99	0.12	0.81	---	0.89	0.84	0.8	---	0.72	0.11
VRC26.25	Susceptibility	0.46	0.4	0.85	0.73	0.75	0.85	0.79	0.77	0.79	0.59	0.87	0.4
VRC07-523-LS + 10-1074	Susceptibility	0.01	0.71	1	0.1	0.9	---	0.95	0.92	0.9	---	0.77	0.12
VRC07-523-LS + 10-1074	Multiple susceptibility	0.02	0.67	0.99	0.12	0.89	---	0.94	0.9	0.88	---	0.78	0.13
VRC07-523-LS + PGDM1400	Susceptibility	0.01	0.65	0.99	0	0.9	---	0.94	0.91	0.89	---	0.59	-0.03
VRC07-523-LS + PGDM1400	Multiple susceptibility	0.01	0.77	0.98	0.04	0.89	---	0.93	0.91	0.88	---	0.64	0.02
VRC07-523-LS + PGT121	Susceptibility	0.01	0.47	1	0.09	0.88	---	0.94	0.9	0.88	---	0.74	0.13
VRC07-523-LS + PGT121	Multiple susceptibility	0.06	0.76	0.98	0.33	0.9	---	0.94	0.92	0.89	---	0.74	0.14
VRC07-523-LS + PGT121 + PGDM1400	Susceptibility	0.01	0.85	0.99	0.08	0.94	---	0.96	0.95	0.93	---	0.72	0.03
VRC07-523-LS + PGT121 + PGDM1400	Multiple susceptibility	0.01	0.8	0.99	0.11	0.93	---	0.96	0.95	0.93	---	0.7	0.04

Table S3. Prediction performance (sensitivity, specificity, positive predictive value [PPV], negative predictive value [NPV], F1 score [harmonic mean of sensitivity and PPV], F0.5 score [weighted harmonic mean of sensitivity and PPV, with more weight given to PPV], accuracy, Matthews correlation coefficient [MCC], area under the receiver operating characteristic curve [AUC], and Brier score) *at the quantile of predicted risk that maximized the MCC* when predicting the binary outcomes for each broadly neutralizing antibody (bnAb) or bnAb regimen from Table 1, Related to Table 1. Susceptibility is defined as (combination) IC_{80} < 1 μg/ml, where for *J* bnAbs, combination $IC_{80} = \left(\sum_{j=1}^{J} IC_{80,j}^{-1}\right)^{-1}$ and multiple susceptibility is defined as $IC_{80,j}$ < 1 for at least 1 bnAb (where applicable).

**bnAb (or bnAb regimen)**	**Outcome**	**Quantile**	**Cutoff**	**Sensitivity**	**Specificity**	**PPV**	**NPV**	**F1**	**F0.5**	**Accuracy**	**MCC**	**AUC**	**Brier**
VRC01	Susceptibility	0.79	0.53	0.41	0.92	0.76	0.71	0.53	0.65	0.72	0.39	0.74	0.19
PGT121	Susceptibility	0.43	0.38	0.88	0.7	0.72	0.87	0.79	0.74	0.78	0.58	0.84	0.36
VRC01/PGDM1400/ 10e8v4	Susceptibility	0.11	0.72	0.95	0.43	0.88	0.81	0.91	0.89	0.85	0.49	0.79	0.2
VRC07-523-LS	Susceptibility	0.16	0.72	0.9	0.4	0.85	0.51	0.87	0.86	0.79	0.33	0.72	0.11
VRC26.25	Susceptibility	0.53	0.5	0.78	0.81	0.79	0.8	0.78	0.79	0.79	0.59	0.87	0.4
VRC07-523-LS + 10-1074	Susceptibility	0.08	0.83	0.95	0.42	0.93	0.47	0.94	0.94	0.9	0.39	0.77	0.12
VRC07-523-LS + 10-1074	Multiple susceptibility	0.23	0.86	0.82	0.61	0.94	0.33	0.87	0.91	0.79	0.34	0.78	0.13
VRC07-523-LS + PGDM1400	Susceptibility	0.26	0.88	0.75	0.44	0.92	0.15	0.82	0.88	0.72	0.12	0.59	-0.03
VRC07-523-LS + PGDM1400	Multiple susceptibility	0.36	0.89	0.67	0.58	0.93	0.18	0.77	0.86	0.66	0.17	0.64	0.02
VRC07-523-LS + PGT121	Susceptibility	0.1	0.79	0.94	0.36	0.91	0.52	0.92	0.91	0.86	0.35	0.74	0.13
VRC07-523-LS + PGT121	Multiple susceptibility	0.11	0.79	0.94	0.44	0.92	0.47	0.93	0.92	0.87	0.38	0.74	0.14
VRC07-523-LS + PGT121 + PGDM1400	Susceptibility	0.17	0.92	0.85	0.51	0.96	0.23	0.9	0.93	0.83	0.26	0.72	0.03
VRC07-523-LS + PGT121 + PGDM1400	Multiple susceptibility	0.1	0.89	0.92	0.35	0.95	0.3	0.93	0.94	0.88	0.25	0.7	0.04

Table S4 is provided as a separate Excel file.

Table S5. Prediction performance for each broadly neutralizing antibody (bnAb) or bnAb regimen from Table 1, Related to the STAR Methods. For each SLAPNAP run, performance is measured using cross-validated R-squared (CV-R^2) for continuous IC_{80}. For a bnAb regimen with J bnAbs, combination IC_{80} = $\left(\sum_{j=1}^{J} \mathrm{IC}_{80,j}^{-1}\right)^{-1}$.

**bnAb (or bnAb regimen)**	**Prediction Performance IC_{80}**
VRC01	0.345
PGT121	0.571
VRC07-523-LS	0.193
VRC26.25 (CAP256)	0.53
VRC07-523-LS + 10-1074	0.319
VRC07-523-LS + PGT121	0.316
VRC07-523-LS + PGDM1400	0.255
VRC07-523-LS + PGT121 + PGDM1400	0.181
VRC01/PGDM1400/10e8v4	0.254

Table S6. Prediction performance for each broadly neutralizing antibody (bnAb) or bnAb regimen from Table 1, Related to the STAR Methods. For each SLAPNAP run, performance is measured using cross-validated AUC (CV-AUC) for susceptibility, defined as (combination) IC_{80} < 1 μg/ml, where for *J* bnAbs, combination $IC_{80} = \left(\sum_{j=1}^{J} \mathrm{IC}_{80,j}^{-1}\right)^{-1}$; and multiple susceptibility, defined as $IC_{80,j} < 1$ for at least 1 bnAb (where applicable).

	Prediction Performance	
**bnAb (or bnAb regimen)**	**(Combination) Susceptibility**	**Multiple Susceptibility**
VRC01	0.744	-
PGT121	0.85	-
VRC07-523-LS	0.728	-
VRC26.25 (CAP256)	0.867	-
VRC07-523-LS + 10-1074	0.783	0.784
VRC07-523-LS + PGT121	0.768	0.781
VRC07-523-LS + PGDM1400	0.638	0.669
VRC07-523-LS + PGT121 + PGDM1400	0.73	0.708
VRC01/PGDM1400/10e8v4	0.81	-

Table S7. HXB2-reference position in HIV-1 Env gp120 identified as being important for predicting sensitivity to VRC01, along with the putative sensitive residue at the given position and the proportion of AMP trial placebo arm participants with the putative sensitive residue at the position, Related to the STAR Methods.

HXB2 Position	Putative Sensitive Residue	Prop. AMP Placebo Participants with Sensitive Residue ($\gamma_{0,j}$)
60	A	0.852
142	T	0.173
144	N	0.098
147	T	0.024
170	Q	0.198
230	Not D	0.556
279	N	0.383
280	N	0.975
317	F	0.840
365	S	0.815
429	E	0.506
456	R	0.963
458	G	1
459	G	0.951
471	G	0.580

Table S8. Values of $PE(S_{230} = 1)$ based on differing values of $PE(S_{230} = 0)$, Related to the STAR Methods. These values are guaranteed to satisfy the constraint on overall PE provided in Equation (S3).

$PE(S_{230} = 0)$	$PE(S_{230} = 1)$
0	0.89
0.233	0.86
0.466	0.81
0.7	0.7

Supplemental References

S1. Gilbert, P. B., Huang, Y., deCamp, A. C., Karuna, S., Zhang, Y., Magaret, C. A., Giorgi, E. E., Korber, B., Edlefsen, P. T., Rossenkhan, R. et al. (2022). Neutralization titer biomarker for antibody-mediated prevention of HIV-1 acquisition. Nat. Med. 28, 1924-1932.

S2. Mayer, K. H., Seaton, K. E., Huang, Y., Grunenberg, N., Isaacs, A., Allen, M., Ledgerwood, J. E., Frank, I., Sobieszczyk, M. E., Baden, L. R. et al. (2017). Safety, pharmacokinetics, and immunological activities of multiple intravenous or subcutaneous doses of an anti-HIV monoclonal antibody, VRC01, administered to HIV-uninfected adults: Results of a phase 1 randomized trial. PLoS Med. 14, e1002435.

S3. Corey, L., Gilbert, P. B., Juraska, M., Montefiori, D. C., Morris, L., Karuna, S. T., Edupuganti, S., Mgodi, N. M., deCamp, A. C., Rudnicki, E. et al. (2021). Two randomized trials of neutralizing antibodies to prevent HIV-1 acquisition. N. Engl. J. Med. 384, 1003-1014.
